# Adjusting for treatment selection in phase II/III clinical trials with time to event data

**DOI:** 10.1002/sim.9606

**Published:** 2022-11-23

**Authors:** Josephine N. Khan, Peter K. Kimani, Ekkehard Glimm, Nigel Stallard

**Affiliations:** ^1^ MRC Biostatistics Unit University of Cambridge Cambridge UK; ^2^ Warwick Medical School University of Warwick Coventry UK; ^3^ Novartis Campus Novartis Pharma AG Basel Switzerland

**Keywords:** adaptive seamless design, multi‐arm multi‐stage, point estimation, survival data

## Abstract

Phase II/III clinical trials are efficient two‐stage designs that test multiple experimental treatments. In stage 1, patients are allocated to the control and all experimental treatments, with the data collected from them used to select experimental treatments to continue to stage 2. Patients recruited in stage 2 are allocated to the selected treatments and the control. Combined data of stage 1 and stage 2 are used for a confirmatory phase III analysis. Appropriate analysis needs to adjust for selection bias of the stage 1 data. Point estimators exist for normally distributed outcome data. Extending these estimators to time to event data is not straightforward because treatment selection is based on correlated treatment effects and stage 1 patients who do not get events in stage 1 are followed‐up in stage 2. We have derived an approximately uniformly minimum variance conditional unbiased estimator (UMVCUE) and compared its biases and mean squared errors to existing bias adjusted estimators. In simulations, one existing bias adjusted estimator has similar properties as the practically unbiased UMVCUE while the others can have noticeable biases but they are less variable than the UMVCUE. For confirmatory phase II/III clinical trials where unbiased estimators are desired, we recommend the UMVCUE or the existing estimator with which it has similar properties.

## 1 INTRODUCTION

Phase II/III clinical trials are efficient since they can answer objectives of phases II and III in a single two‐stage trial.^1^, ^2^, ^3^, ^4^ In stage 1, patients are allocated to a control and multiple experimental treatments. The stage 1 data are used to determine the experimental treatment(s) that, together with the control, continue to stage 2. A phase II/III clinical trial is efficient because data of stage 1 and stage 2 are combined to perform a confimatory phase III analysis. However, failing to adjust for treatment selection in the analysis of phase II/III clinical trials can lead to substantial biases.^5^ For hypothesis testing, several methods that adjust for treatment selection exist for normally distributed data^1^, ^2^, ^6^, ^7^ and time to event data.^8^, ^9^, ^10^ In this paper, we focus on point estimation.

When outcomes are normally distributed, several point estimators that adjust for using stage 1 data for treatment selection have been proposed. The statistical methods used to develop the estimators can be grouped into three based on statistical techniques used to adjust for treatment selection. For the first, estimated bias is subtracted from the naive point estimate.^11^ The estimates for the second and third methods are computed using expressions for a shrinkage estimator^12^ and a uniformly minimum variance conditional estimator (UMVCUE).^13^, ^14^, ^15^, ^16^, ^17^ These results extend asymptotically in cases where the responses uniquely pertain to one stage of the design such as binary response variables. In trials with time‐to‐event‐endpoints, however, observation of individuals across stages poses an additional complication.

There are two main features that make extension of estimators for normally distributed outcomes to TTE outcomes complex. Treatment selection may be based on treatment differences that are correlated so that there is need to determine the joint distribution of these differences that do not have a diagonal covariance matrix. The covariance matrix for TTE outcomes is not as well established in literature and as straightforward as for normally distributed outcomes and so it is important to describe it in detail. Secondly, it is more efficient for patients without events in stage 1 to be followed further in stage 2. However, this induces correlation between stage 1 and stage 2 data. Brückner et al^18^ extended a shrinkage estimator and an estimator that involves estimating bias and subtracting it from the naive estimator to TTE. Their conclusion was that the two estimators over‐correct for bias and only the shrinkage estimator can have smaller mean squared error (MSE) than the naive estimator. Therefore, there is need to explore more estimators for phase II/III clinical trials with TTE data.

We have extended the UMVCUE for normally distributed outcomes to derive a new approximately UMVCUE for TTE outcomes and have compared the new estimator to the estimators by Brückner et al. We have derived expressions that encompass several treatment selection rules and that include the possibility of stopping for futility, which give more general estimators compared to Brückner et al who considered a single selection rule and assumed that the trial always continued to stage 2. Also, unlike Brückner et al, we have proposed how to reduce the impact of correlation between stage 1 and stage 2 data and have incorporated these in the estimators.

## 2 SETTING AND BIAS OF NAIVE ESTIMATOR

### 2.1 Setting and combining information from different stages

In stage 1, K≥2 experimental treatments are compared to a common control. We denote the true log hazard ratio (HR) for the comparison between control and experimental treatment j (j=1,…,K) by $\theta_{j}$, with $\theta_{j}<0$ indicating treatment j is superior to the control. As we will describe in this section, we will compute pairwise log HRs estimates based on the score statistics derived from the partial likelihood. Consequently, we assume proportional hazards for the comparison between the control and each experimental treatment.

The trial starts recruitment at time $t_{0}$. We assume that there is a prefixed condition of when the interim analysis is performed. The requirement for the condition is described in Section 2.2. For example, as in the simulation study in Section 6, interim analysis may be performed when a prefixed total number of events is observed from all treatments including the control. There are alternatives such as fixing the calendar time of interim analysis, which we use in Section 5, but these are less common in practice. We refer to patients whose data (including censored observations) are used in the interim analysis as stage 1 patients. Like Kimani et al,^19^ we first consider basing estimation on the logrank score statistic and use similar notation. We denote the score statistic and the Fisher information for comparing treatment j (j=1,…,K) to the control using stage 1 data by $S_{1,j}$ and $V_{1,j}$, respectively. Asymptotically, $S_{1,j}∼N(\theta_{j}V_{1,j},V_{1,j})$ so that ${\hat{\theta }}_{1,j}$ defined as $S_{1,j}/V_{1,j}$ is asymptotically $N(\theta_{j},\sigma_{1,j}^{2})$, where $\sigma_{1,j}^{2}=1/V_{1,j}$.^20^, ^21^ We demonstrate how to extract the logrank score statistic and the Fisher's information for the example data in Section 5 using the R statistical package in the code in the repository “https://github.com/KimaniPK/Treatment‐Selection‐with‐TTE‐data”.^22^ Their expressions can be found in textbooks such as Jennison and Turnbull (Chapter 3.7 and Chapter 13).^23^

The patients recruited in stage 2, who we refer to as stage 2 patients, are randomized to the control and the experimental treatments that are selected for further testing based on stage 1 results. Rules for selecting treatments are described in Section 2.5. We assume a prefixed condition for how long to follow stage 2 patients. The requirement for the condition is described in Section 2.2. As before, this could be until a prefixed number of events are observed from the stage 2 patients or the less common conditions such as at a prespecified calendar date. Also, in stage 2, we assume that there is additional follow up of stage 1 patients from the control and the selected experimental treatments that did not have events during the interim analysis. Let 𝒮 denote the set of indices for the selected treatments. We denote the score statistic and the Fisher information for comparing experimental treatment j (j∈𝒮) to the control based on all data from patients recruited in both stage 1 and stage 2 by $S_{j}$ and $V_{j}$, respectively. The estimators that adjust for treatment selection that we will consider in Section 3 assume that the test statistics attributed to data collected in stage 2 are independent of the test statistics based on stage 1 data, the so called independent increment structure. We describe below in Section 2.2 how to get an approximate independent increment structure.^23^, ^24^, ^25^ Assuming that the independent increment structure holds, asymptotically $S_{j}∼N(\theta_{j}V_{j},V_{j})$ so that asymptotically ${\hat{\theta }}_{j}$ defined as $S_{j}/V_{j}$ is $N(\theta_{j},\sigma_{j}^{2})$, where $\sigma_{j}^{2}=1/V_{j}$. Furthermore, asymptotically, the stage 2 increment $S_{j}-S_{1,j}∼N(\theta_{j}(V_{j}-V_{1,j}),V_{j}-V_{1,j})$, and consequently, asymptotically ${\hat{\theta }}_{2,j}$ defined as $\left(S_{j}-S_{1,j})/(V_{j}-V_{1,j}\right)$ is $N(\theta_{j},\sigma_{2,j}^{2})$, where $\sigma_{2,j}^{2}=1/(V_{j}-V_{1,j})$.

### 2.2 Approximate independent structure

In stage 2, data are collected from two sets of patients namely (i) the stage 2 patients and (ii) the stage 1 patients without events prior to the interim analysis. To have independent increment structure, for each of the two sets of patients, we aim that data collected from them in stage 2 are independent of stage 1 data. Stage 2 patients are new patients recruited after the interim analysis. Therefore, their data are independent of stage 1 data if the number of stage 2 patients and either the number of events from them or calendar length of how long to follow them are set independent of stage 1 data. This is what we have proposed in Section 2.1.

Patients in the other set, that is, stage 1 patients without events up to the interim analysis that are followed in stage 2, contribute to both stage 1 data and stage 2 data. Furthermore, these patients are followed in stage 2 because of the selection made using stage 1 data. Consequently, following in stage 2 patients without events during the interim analysis can induce correlation between stage 1 and stage 2 data. This corresponds to the second complexity with TTE data stated in the introduction. To approximately achieve independence between data collected in stage 1 and stage 2, we will use the strategy by Jenkins et al.^26^ They propose prespecifiying a rule for how long stage 1 patients are followed in stage 2. This makes $V_{j}-V_{1,j}$ independent of stage 1 data but does not necessarily make $S_{j}-S_{1,j}$ independent of stage 1 data because of treatment selection and hence the reason that the proposal by Jenkins et al is an approximation of the independent structure. In Sections 5 and 6, we will fix calendar duration for how long stage 1 patients are followed in stage 2. Alternatives include fixing the number of events in stage 2 from stage 1 patients.

We have set conditions for when the interim analysis is performed, how long to follow patients recruited in stage 2 and how long to follow stage 1 patients after the interim analysis. This is a practical design to achieve approximate independent increment structure. The bias adjusted estimators we will consider are appropriate for any design where independent increment structure can be assumed. The correlation between stage 1 and stage 2 data depends on several factors such as the true hazard functions, the recruitment rate and the true log hazard ratios. In simulations, the Jenkins et al approach achieved approximate independence between stage 1 and stage 2 estimates when these factors were varied in two‐stage clinical trials with subpopulation selection.^19^

### 2.3 Joint distribution

We first consider the case that treatment one only is selected, that is, 𝒮={1}. We describe the extension to any 𝒮 at the end of the section. The estimators developed require knowledge of the joint distribution for ${\left({\hat{\theta }}_{1,1},{\hat{\theta }}_{1,2},\ldots ,{\hat{\theta }}_{1,K},{\hat{\theta }}_{2,1}\right)}^{\prime }$. We assume that ${\hat{\theta }}_{2,1}$ as defined in Section 2.1 achieves independent increment structure so that ${\hat{\theta }}_{2,1}$ is independent of the K stage 1 estimates ${\hat{\theta }}_{1,1},\ldots ,{\hat{\theta }}_{1,K}$. Thus, from Section 2.1, we only need to give the expression for $\text{Cov}({\hat{\theta }}_{1,i},{\hat{\theta }}_{1,j})$, which we denote by $q_{ij}$, to have the joint distribution for ${\left({\hat{\theta }}_{1,1},{\hat{\theta }}_{1,2},\ldots ,{\hat{\theta }}_{1,K},{\hat{\theta }}_{2,1}\right)}^{\prime }$ fully defined. Di Scala and Glimm^10^, ^27^ give the expression for $\text{Cov}({\hat{\theta }}_{1,i}\sqrt{V_{1,i}},{\hat{\theta }}_{1,j}\sqrt{V_{1,j}})$ (i≠j,i,j=1,…,K) from which it is straightforward to derive the expression for $\text{Cov}({\hat{\theta }}_{1,i},{\hat{\theta }}_{1,j})$. Consider the control and experimental treatments i and j. Suppose that there are D events from the three arms in stage 1. For simplicity in the expression for $\text{Cov}({\hat{\theta }}_{1,i},{\hat{\theta }}_{1,j})$, we assume that the events occur at unique times. At event time $t_{d}$ (d=1,…,D), let $r_{d,c}$, $r_{d,i}$ and $r_{d,j}$ denote the number of patients at risk for the control and experimental treatments i and j, respectively. For $t_{d}$ (d=1,…,D) and experimental treatment i we define $p_{d,i}=0$ if the event came from experimental treatment j (j≠i) and $p_{d,i}=r_{d,i}/(r_{d,c}+r_{d,i})$ otherwise. For experimental treatment j, $p_{d,j}$ is defined similarly. Following Di Scala and Glimm^10^, ^27^ $q_{ij}=\text{Cov}({\hat{\theta }}_{1,i},{\hat{\theta }}_{1,j})=\sigma_{1,i}^{2}\sigma_{1,j}^{2}\sum_{d=1}^{D}p_{d,i}p_{d,j}$. Consequently,

(1) $$\left(\begin{array}{c} {\hat{\theta }}_{1,1}\\ {\hat{\theta }}_{1,2}\\ \cdot \\ \cdot \\ \cdot \\ {\hat{\theta }}_{1,K}\\ {\hat{\theta }}_{2,1} \end{array}\right)∼MVN\left(\begin{array}{c} \left(\begin{array}{c} \theta_{1}\\ \theta_{2}\\ \cdot \\ \cdot \\ \cdot \\ \theta_{K}\\ \theta_{1} \end{array}\right)\;,\left(\begin{array}{ccccccc} \sigma_{1,1}^{2} & q_{12} & \cdot & \cdot & \cdot & q_{1K} & 0\\ q_{12} & \sigma_{1,2}^{2} & \cdot & \cdot & \cdot & q_{2K} & 0\\ \cdot & \cdot & \cdot & & & & \cdot \\ \cdot & \cdot & & \cdot & & & \cdot \\ \cdot & \cdot & & & \cdot & & \cdot \\ q_{1K} & q_{2K} & \cdot & \cdot & \cdot & \sigma_{1,K}^{2} & 0\\ 0 & 0 & \cdot & \cdot & \cdot & 0 & \sigma_{2,1}^{2} \end{array}\right) \end{array}\right).$$

An alternative way to obtain the joint distribution above is to use transformation of random variables to transform the joint distribution derived by Di Scala and Glimm.^10^ It is straightforward to extend the joint distribution to have multiple experimental treatments in stage 2. However, we did not give this distribution as it requires additional notation, which is not required in the estimators in this paper since only stage 1 data determine if a treatment proceeds to stage 2.

### 2.4 Joint distribution based on Cox's proportional hazards model

An alternative to basing estimation of ${\left({\hat{\theta }}_{1,1},\ldots ,{\hat{\theta }}_{1,K}\right)}^{\prime }$ and ${\hat{\theta }}_{2,j}$ (j∈𝒮) on logrank score statistics is using Cox's proportional hazards models. Utilizing all stage 1 data, we propose obtaining stage 1 estimates by fitting a Cox's proportional hazards model (stage 1 model) that has K predictors corresponding to indicators for the K experimental treatments.^18^ Log HR estimates obtained are different to those of fitting K Cox's proportional hazards models comparing the control to each experimental treatment. However, the advantage of using a single model is that log HR estimates and the variance‐covariance matrix can be obtained from standard statistical packages making practical implementation easier. We use the same notation as Section 2.1 with ${\hat{\theta }}_{1,j}$ and $\sigma_{1,j}^{2}$ (j=1,…,K) denoting the estimate and variance corresponding to the estimator for $\theta_{j}$ from stage 1 model and $q_{ij}$ (i≠j,i,j=1,…,K) the covariance for the estimators for $\theta_{i}$ and $\theta_{j}$. For j∈𝒮, a Cox's proportional hazards model (stage 2 model) is fitted using all stage 1 data and stage 2 data collected from the control arm and experimental treatment j only. The follow up in stage 2 of stage 1 patients from the control and treatment j without events in stage 1 is as described in Section 2.2. The log HR estimate and variance corresponding to treatment j obtained using stage 2 model are denoted by ${\hat{\theta }}_{j}$ and $\sigma_{j}^{2}$, respectively. To obtain the approximately independent stage 2 estimate and its variance, we follow Jennison and Turnbull (pages 262‐264).^21^ We compute $V_{1,j}=1/\sigma_{1,j}^{2}$, $S_{1,j}={\hat{\theta }}_{1,j}V_{1,j}$, $V_{j}=1/\sigma_{j}^{2}$ and $S_{j}={\hat{\theta }}_{j}V_{j}$. The stage 2 approximate independent estimate is ${\hat{\theta }}_{2,j}=(S_{j}-S_{1,j})/(V_{j}-V_{1,j})$ and its variance is $\sigma_{2,j}^{2}=1/(V_{j}-V_{1,j})$.

Since we use the same notation for estimation based on the logrank score statistic and estimation based on the Cox's proportional hazards model, expression (1) is the common form of the joint distributions for estimators based on the two models. We emphasize that although the two models have distributions with a common expression that arise from sharing notation, in general, the parameter values for the joint distributions corresponding to the two models are different.

### 2.5 Treatment selection rules

Conditional on a prefixed condition of when the interim analysis is performed which may be based on $\left(V_{1,1},\ldots ,V_{1,K}\right)$, we will consider selection rules where it is possible to use the observed vector ${\left({\hat{\theta }}_{1,1},\ldots ,{\hat{\theta }}_{1,K}\right)}^{\prime }$ only to determine the treatment(s) to be selected. Based on the observed stage 1 estimates, the estimators we will describe will require describing the region where a treatment is selected by either specifying the limits for all stage 1 treatments' effects or the limits for the effect of a selected treatment conditional on the effects for the other K−1 treatments. We describe some treatment selection rules. One rule is to select the best treatment based on observed stage 1 log HR. For this rule, based on limits of all treatments' effects, experimental treatment j (j∈{1,…,K}) is selected if $-\infty <{\hat{\theta }}_{1,j}<\infty$ and ${\hat{\theta }}_{1,j}<{\hat{\theta }}_{1,j^{\prime }}<\infty$ ($j^{\prime }\neq j,\;j^{\prime }=1,\ldots ,K$). For all selection rules, we will denote the lower and upper bounds for the effect of a selected treatment j (j∈{1,…,K}) conditional on the effects for the other K−1 treatment effects by $L_{j}$ and $W_{j}$, respectively. For the aforementioned selection rule, $L_{j}=-\infty$ and $W_{j}=\min\left(\{{\hat{\theta }}_{1,1},\ldots ,{\hat{\theta }}_{1,K}\}\setminus {\hat{\theta }}_{1,j}\right)$. Some other selection rules are summarized in Table 1. We have given examples of selection rules where $L_{j}=-\infty$ always. We summarize Bowden and Glimm^14^ selection rule where sometimes $L_{j}\neq -\infty$ in the Supplementary Material because Table 1 format does not suit this selection rule.

**TABLE 1:** Summary of common selection rules

	Limits for individual treatments' effects		A selected treatment upper bound
Selected experimental treatment(s)	j∈𝒮	j∈𝒮, $j^{\prime }\in {𝒮}^{c}$	$\left(W_{j}\right)$ conditional on other effects^a^
All treatments with log HR ≤b	$-\infty <{\hat{\theta }}_{1,j}\leq b$	$b<{\hat{\theta }}_{1,j^{\prime }}<\infty$	b
All treatments with P‐values^b^ ≤a	$-\infty <{\hat{\theta }}_{1,j}\leq \frac{\Phi^{-1}(a)}{\sqrt{V_{1,j}}}$	$\frac{\Phi^{-1}(a)}{\sqrt{V_{1,j^{\prime }}}}<{\hat{\theta }}_{1,j^{\prime }}<\infty$	$\frac{\Phi^{-1}(a)}{\sqrt{V_{1,j}}}$
Treatment with smallest log HR	$-\infty <{\hat{\theta }}_{1,j}<\infty$	${\hat{\theta }}_{1,j}<{\hat{\theta }}_{1,j^{\prime }}<\infty$	$\min\left(\{{\hat{\theta }}_{1,1},\ldots ,{\hat{\theta }}_{1,K}\}\setminus {\hat{\theta }}_{1,j}\right)$
Treatment with smallest P‐value^b^	$-\infty <{\hat{\theta }}_{1,j}<\infty$	${\hat{\theta }}_{1,j}\sqrt{\frac{V_{1,j}}{V_{1,j^{\prime }}}}<{\hat{\theta }}_{1,j^{\prime }}<\infty$	$\min\left(\left\{{\hat{\theta }}_{1,1}^{I_{j}},\ldots ,{\hat{\theta }}_{1,K}^{I_{j}}\right\}\setminus {\hat{\theta }}_{1,j}^{I_{j}}\right)$
Treatment with smallest log HR ≤b	$-\infty <{\hat{\theta }}_{1,j}\leq b$	${\hat{\theta }}_{1,j}<{\hat{\theta }}_{1,j^{\prime }}<\infty$	$\min\left(\{b,{\hat{\theta }}_{1,1},\ldots ,{\hat{\theta }}_{1,K}\}\setminus {\hat{\theta }}_{1,j}\right)$
Treatment with smallest P‐value^b^ ≤a	$-\infty <{\hat{\theta }}_{1,j}\leq \frac{\Phi^{-1}(a)}{\sqrt{V_{1,j}}}$	${\hat{\theta }}_{1,j}\sqrt{\frac{V_{1,j}}{V_{1,j^{\prime }}}}<{\hat{\theta }}_{1,j^{\prime }}<\infty$	$\min\left(\left\{a^{I_{j}},{\hat{\theta }}_{1,1}^{I_{j}},\ldots ,{\hat{\theta }}_{1,K}^{I_{j}}\right\}\setminus {\hat{\theta }}_{1,j}^{I_{j}}\right)$

*Note*: ${𝒮}^{c}=\{1,\ldots ,K\}\setminus 𝒮$.

a For all selection rules, lower bound $L_{j}=-\infty$ and for j and $j^{\prime }$ ($j,j^{\prime }=1,\ldots ,K$), we define ${\hat{\theta }}_{1,j^{\prime }}^{I_{j}}={\hat{\theta }}_{1,j^{\prime }}\sqrt{V_{1,j^{\prime }}/V_{1,j}}$ and $a^{I_{j}}=\Phi^{-}(a)/\sqrt{V_{1,j}}$.

b These are pairwise one‐sided P‐values.

### 2.6 Naive estimation

We consider ${\hat{\theta }}_{j}$ (j∈𝒮) to be the naive estimator for $\theta_{j}$. Note that from the definitions for ${\hat{\theta }}_{1,j}$, ${\hat{\theta }}_{j}$ and ${\hat{\theta }}_{2,j}$ in Section 2.1, ${\hat{\theta }}_{j}$ can be expressed as

(2) $${\hat{\theta }}_{j}=\frac{\sigma_{2,j}^{2}{\hat{\theta }}_{1,j}+\sigma_{1,j}^{2}{\hat{\theta }}_{2,j}}{\sigma_{1,j}^{2}+\sigma_{2,j}^{2}}.$$

From Sections 2.1 and 2.2, we assume conditions for how long to follow patients are preset such that there is approximate independent increment structure. Therefore, from expression (2), ${\hat{\theta }}_{j}$ is the sum of two parts that are approximately independent. Furthermore, since there is no selection in stage 2, we assume that ${\hat{\theta }}_{2,j}$ is unbiased for $\theta_{j}$. Consequently, while deriving the expression for the bias for ${\hat{\theta }}_{j}$ and while adjusting for treatment selection in Section 3, we will assume that data collected in stage 1 and those collected in stage 2 data are not correlated so that the biases in naive estimates are due to treatment selection at the interim analysis only. These assumptions are reasonable since from the simulations results in Section 6.2, the biases for the approximately unbiased estimator derived in Section 4 are small.

Define $t_{j}=V_{1,j}/V_{j}$ and let $1_{𝒮}$ and Prob(𝒮), denote the indicator and probability for the event that the set of indices for the selected treatments is 𝒮, respectively. From expression (2), the bias for ${\hat{\theta }}_{j}$ can be expressed as

(3) $$\text{Bias}({\hat{\theta }}_{j})=t_{j}\left(\frac{E\left[{\hat{\theta }}_{1,j}1_{𝒮}\right]}{\text{Prob}(𝒮)}-\theta_{j}\right).$$

For $j^{\prime }\in {𝒮}^{c}$, we take ${\hat{\theta }}_{1,j^{\prime }}$ to be the naive estimator for $\theta_{j^{\prime }}$. The bias for ${\hat{\theta }}_{1,j^{\prime }}$ is given by

(4) $$\text{Bias}({\hat{\theta }}_{1,j^{\prime }})=\left(E\left[{\hat{\theta }}_{1,j^{\prime }}1_{𝒮}\right]/\text{Prob}(𝒮)\right)-\theta_{j^{\prime }}.$$

Simple to compute expressions for Prob(𝒮), $E\left[{\hat{\theta }}_{1,j}1_{𝒮}\right]$ and $E\left[{\hat{\theta }}_{1,j^{\prime }}1_{𝒮}\right]$ in expressions (3) and (4) can be obtained if the selection rule is known. Let us consider the case of selecting an experimental treatment if its corresponding stage 1 observed log HR is below b and it is smallest among the K experimental treatments (fifth selection rule in Table 1). For demonstration, we suppose that experimental treatment 1 is selected, that is, 𝒮={1}. Let A be the K×K matrix given in the Supplementary Material where the first element in column one is 1 while all the other elements in column one are equal to −1. For column i (i=2,…,K), the $i^{\text{th}}$ element is equal to 1 while the other elements are all equal to zero. Let ${\hat{\theta }}_{1}$ and ${\hat{\delta }}_{1}$ denote the vectors ${\left({\hat{\theta }}_{1,1},\ldots ,{\hat{\theta }}_{1,K}\right)}^{\prime }$ and ${\left({\hat{\delta }}_{1,1},\ldots ,{\hat{\delta }}_{1,K}\right)}^{\prime }$, respectively, where ${\hat{\delta }}_{1}=A{\hat{\theta }}_{1}$ so that ${\hat{\delta }}_{1,1}={\hat{\theta }}_{1,1}$ and ${\hat{\delta }}_{1,j}={\hat{\theta }}_{1,j}-{\hat{\theta }}_{1,1}$ (j=2,…,K). Then

(5) $$\text{Prob}(𝒮)=\int_{-\infty }^{b}\int_{{\hat{\theta }}_{1,1}}^{\infty }\cdot \cdot \cdot \int_{{\hat{\theta }}_{1,1}}^{\infty }f({\hat{\theta }}_{1,1},\ldots ,{\hat{\theta }}_{1,K})d{\hat{\theta }}_{1,K}\cdot \cdot \cdot d{\hat{\theta }}_{1,2}d{\hat{\theta }}_{1,1}=\int_{-\infty }^{b}\int_{0}^{\infty }\cdot \cdot \cdot \int_{0}^{\infty }f({\hat{\delta }}_{1,1},\ldots ,{\hat{\delta }}_{1,K})d{\hat{\delta }}_{1,K}\cdot \cdot \cdot d{\hat{\delta }}_{1,2}d{\hat{\delta }}_{1,1},$$

where $f({\hat{\theta }}_{1,1},\ldots ,{\hat{\theta }}_{1,K})$ and $f({\hat{\delta }}_{1,1},\ldots ,{\hat{\delta }}_{1,K})$ are the densities for ${\left({\hat{\theta }}_{1,1},\ldots ,{\hat{\theta }}_{1,K}\right)}^{\prime }$ and ${\left({\hat{\delta }}_{1,1},\ldots ,{\hat{\delta }}_{1,K}\right)}^{\prime }$, respectively. Let $\theta ={\left(\theta_{1},\ldots ,\theta_{K}\right)}^{\prime }$ and $\sum_{{\hat{\theta }}_{1}}$ be the matrix obtained by excluding the last column and row in the variance covariance matrix in expression (1). Note that ${\hat{\theta }}_{1}$ is $MVN(\theta ,\sum_{{\hat{\theta }}_{1}})$ and since ${\hat{\delta }}_{1}$ is a linear combination of ${\hat{\theta }}_{1}$, it is $MVN(\delta ,\sum_{{\hat{\delta }}_{1}})$, where δ=Aθ and $\sum_{{\hat{\delta }}_{1}}=A\sum_{{\hat{\theta }}_{1}}A^{\prime }$. Computing Prob(𝒮) using the density for ${\hat{\delta }}_{1}$ is computational easier and can be done using standard statistical programs, such as “pmvnorm” function in “mvtnorm” package^28^ in R,^29^ because limits of integration are not dependent on the random variables. From the properties of taking expectation, $E\left[{\hat{\theta }}_{1,1}1_{𝒮}\right]=E\left[{\hat{\delta }}_{1,1}1_{𝒮}\right]$, where

(6) $$E\left[{\hat{\delta }}_{1,1}1_{𝒮}\right]=\int_{-\infty }^{b}\int_{0}^{\infty }\cdot \cdot \cdot \int_{0}^{\infty }{\hat{\delta }}_{1,1}f({\hat{\delta }}_{1,1},\ldots ,{\hat{\delta }}_{1,K})d{\hat{\delta }}_{1,K}\cdot \cdot \cdot d{\hat{\delta }}_{1,2}d{\hat{\delta }}_{1,1},$$

For j (j=2,…,K), from the transformation ${\hat{\delta }}_{1,j}={\hat{\theta }}_{1,j}-{\hat{\theta }}_{1,1}$ and properties of expectation, $E[{\hat{\theta }}_{1,j}1_{𝒮}]=E\left[{\hat{\delta }}_{1,j}1_{𝒮}\right]+E\left[{\hat{\theta }}_{1,1}1_{𝒮}\right]$. Note that $E\left[{\hat{\delta }}_{1,j}1_{𝒮}\right]$ (j=2,…,K) is expression (6) with ${\hat{\delta }}_{1,1}$ replaced with ${\hat{\delta }}_{1,j}$. Let $e_{j}$ (j=1,…,K) denote the row vector with $j^{th}$ element equal to one and other elements equal to zero. Following Kan and Robotti,^30^ a simple to compute expression for $E\left[{\hat{\delta }}_{1,j}1_{𝒮}\right]$ (j=1,…,K) is

(7) $$E\left[{\hat{\delta }}_{1,j}1_{𝒮}\right]=\delta_{j}\text{Prob}(𝒮)+e_{j}\sum_{{\hat{\delta }}_{1}}c,$$

where c is a column vector described in the Supplementary Material. Note that from expressions (2), (4), and (7), the bias of stage 1 estimate ${\hat{\theta }}_{1,1}$ is $e_{1}\sum_{{\hat{\delta }}_{1}}c/\text{Prob}(𝒮)$ and the bias of stage 1 estimate ${\hat{\theta }}_{1,j}$ (j=2,…,K) is $\left(e_{j}\sum_{{\hat{\delta }}_{1}}c+e_{1}\sum_{{\hat{\delta }}_{1}}c)/\text{Prob}(𝒮\right)$. For the other selection rules in Table 1, we give expressions for Prob(𝒮), $\text{Bias}({\hat{\theta }}_{j})$ and $\text{Bias}({\hat{\theta }}_{1,j^{\prime }})$ in the Supplementary Material.

## 3 EXISTING ESTIMATORS THAT ADJUST FOR TREATMENT SELECTION

### 3.1 Bias subtracted estimators

We have derived the expression for the bias of the naive estimator in Section 2.6 and we will observe in Section 6 that biases of the naive estimator are substantial in some scenarios. In this section and Section 3.2, we describe bias adjusted estimators based on Brückner et al.^18^

If bias of a naive estimator was known, this could be subtracted from the naive estimator to obtain an unbiased estimator.^11^, ^31^ However, from the expressions in Section 2.6, bias is a function of the unknown vector θ. One option is to substituted the $j^{th}$ element in θ with its naive estimate defined in Section 2.6 while computing bias.^32^ Let $\hat{\theta }$ denote the vector for the naive estimators and $b_{j}(\hat{\theta })$ bias for $\theta_{j}$ computed as described in Section 2.6 while replacing θ with $\hat{\theta }$. Then the bias adjusted estimate for $\theta_{j}$ (j∈𝒮), which, similar to Kimani et al,^32^ we refer to as a single iteration bias subtracted estimator, is given by

(8) $${\hat{\theta }}_{j,SI}={\hat{\theta }}_{j}-b_{j}(\hat{\theta }).$$

Since naive estimates may have substantial bias, another option is to use an iterative procedure.^11^, ^31^ Let $b_{j}(\theta )$ (j=1,…,K) denote bias for the naive estimator for $\theta_{j}$ and b(θ) the bias vector ${\left(b_{1}(\theta ),\ldots ,b_{K}(\theta )\right)}^{\prime }$. The bias is estimated by solving iteratively $\tilde{\theta }=\hat{\theta }-b(\tilde{\theta })$. Let $b_{j}({\tilde{\theta }}_{i})$ denote the bias of $\theta_{j}$ at iteration i (i=1,2,…). If the solution is achieved at iteration r, for j∈𝒮, the multiple iterations bias subtracted estimator for $\theta_{j}$ is given by

(9) $${\hat{\theta }}_{j,MI}={\hat{\theta }}_{j}-b_{j}({\tilde{\theta }}_{r}).$$

### 3.2 Shrinkage estimator

Hwang^33^ proposed a shrinkage estimator for single stage trials for the setting that is analogous to the case where the statistics corresponding to the true treatment effects $\theta_{1},\ldots ,\theta_{K}$ are uncorrelated and have a common variance. A common normal distribution prior for the K treatment effects is assumed. Hwang obtains the posterior mean expression for the selected treatment, and then replaces the prior treatment effect with average of observed effects from the K treatments. This gives empirical Bayes estimator and Hwang shows how to adjust it to get a shrinkage estimator. Carreras and Brannath^12^ extended the work to obtain a shrinkage estimator for two‐stage trials. It is a weighted mean of the shrinkage estimator based on stage 1 data and the unbiased stage 2 estimate ${\hat{\theta }}_{2,j}$.

Brückner et al^18^ described an extension to the case where, as in our setting, stage 1 estimates are correlated. As in Carreras and Brannath, a shrinkage estimator for stage 1 data is obtained, with the two‐stage shrinkage estimator being the weighted mean of the stage 1 shrinkage estimator and the unbiased stage 2 estimator ${\hat{\theta }}_{2,j}$. Let $I_{K}$ denote a K×K identity matrix. A multivariate normal $MVN(\mu ,\nu^{2}I_{K})$ prior for θ is specified. The posterior mean, after updating the prior with ${\hat{\theta }}_{1}$, is $C{\hat{\theta }}_{1}+(I_{K}-C)\mu$, where $C=I_{K}-\sum_{{\hat{\theta }}_{1}}{\left(\nu^{2}I_{K}+\sum_{{\hat{\theta }}_{1}}\right)}^{-1}$. Let ${\hat{\theta }}_{1,all}$ be the stage 1 log hazard ratio estimate obtained by comparing control to all K experimental treatments taken as a single treatment. The prior mean μ in the posterior mean is replaced by a K‐vector that has all elements equal to ${\hat{\theta }}_{1,all}$. The value of $\nu^{2}$ in C is evaluated using an iterative procedure.^18^, ^34^ The procedure is described in the Supplementary Material. Let ${\hat{\theta }}_{j,SH}^{1}$ denote the stage 1 shrinkage estimator for $\theta_{j}$ and w (0≤w≤1) be a prespecified weight. The two‐stage shrinkage estimator is given by

$${\hat{\theta }}_{j,SH}=w{\hat{\theta }}_{j,SH}^{\left(1\right)}+(1-w){\hat{\theta }}_{2,j}.$$

It is reasonable to choose w based on the expected number of events in each stage.^18^ Let $E_{1}$ denote the preplanned number of events at interim analysis from all treatments including the control and $E_{2}$ the preplanned number of events from all stage 2 patients including from the control. In Section 6, where |𝒮| is fixed, we take $w=E_{1}(|𝒮|+1)/\left(E_{1}(|𝒮|+1)+E_{2}(K+1)\right)$.

## 4 NEW APPROXIMATELY UNIFORMLY MINIMUM VARIANCE CONDITIONAL UNBIASED ESTIMATOR

### 4.1 General principles for obtaining the new estimator

The new approximately uniformly minimum variance conditional unbiased estimator (UMVCUE) is based on the Rao‐Blackwell theorem. The UMVCUE is conditional on the selection rule, observed data and a sufficient and complete statistic. For j∈𝒮, the stage 2 estimator ${\hat{\theta }}_{2,j}$ is approximately unbiased for $\theta_{j}$. Therefore, by Rao‐Blackwell theorem, we obtained the UMVCUE for $\theta_{j}$ by deriving the expression for the expected value for ${\hat{\theta }}_{2,j}$ conditional on a sufficient and complete statistic that is based on stage 1 and stage 2 data. Such an estimator was first proposed in two‐stage designs by Cohen and Sackrowitz.^13^ Following Cohen and Sackrowitz, several UMVCUEs have been derived for several settings^14^, ^15^, ^16^, ^17^, ^35^, ^36^ including the case where selection is based on comparing correlated statistics.^16^, ^17^, ^36^ As in our case, the UMVCUE derived by Robertson et al^36^ is based on correlated stage 1 estimates. Kimani et al^32^ build on Robertson et al^36^ to include stopping for futility. Our new UMVCUE builds on Robertson et al and Kimani et al extending the estimators to time to event data based on the asymptotic distribution given by expression (1) and to consider several selection rules.

### 4.2 Deriving the uniformly minimum variance conditional unbiased estimator

In this section, we give the UMVCUE and the main steps we used to derive it. Let $B_{L}$ and $B_{U}$ denote the bounds for ${\hat{\theta }}_{2,j}$. These bounds are functions of $L_{j}$ and $W_{j}$ and hence from Section 2.5, depend on the selection rule used. Expressions for $B_{L}$ and $B_{U}$ for the selection rules in Table 1 are considered in Section 4.3. For j∈𝒮, the UMVCUE for $\theta_{j}$ is given by

(10) $${\hat{\theta }}_{j,UMV}={\hat{\theta }}_{j}-\frac{\sigma_{2,j}^{2}}{\sigma_{1,j}^{2}+\sigma_{2,j}^{2}}\frac{\phi \left\{\left(B_{U}-{\hat{\theta }}_{j}\right)/\eta_{j}\right\}-\phi \left\{\left(B_{L}-{\hat{\theta }}_{j}\right)/\eta_{j}\right\}}{\Phi \left\{\left(B_{U}-{\hat{\theta }}_{j}\right)/\eta_{j}\right\}-\Phi \left\{\left(B_{L}-{\hat{\theta }}_{j}\right)/\eta_{j}\right\}},$$

where $\eta_{j}=\sigma_{2,j}^{2}/\sqrt{\sigma_{1,j}^{2}+\sigma_{2,j}^{2}}$, and ϕ and Φ correspond to density and distribution functions of a standard normal, respectively.

After establishing the joint distribution given by expression (1), the main steps we used to derive the UMVCUE which we describe in the rest of this section follow Robertson et al.^16^ Let $Q_{𝒮}$ denote the event of obtaining the data that led to 𝒮 being the set of indices corresponding to the selected treatments. Further let $1_{Q_{𝒮}}$ be the indicator for $Q_{𝒮}$ and $K_{Q_{𝒮}}(\theta )$ the probability for $Q_{S}$ given θ. Let P denote the inverse of the variance covarinace matrix $\sum_{{\hat{\theta }}_{1}}$. We first consider the case of selecting one treatment and without loss of generality take 𝒮={1}. The joint density for ${\left({\hat{\theta }}_{1},{\hat{\theta }}_{2,1}\right)}^{\prime }$ is given by

$$f_{𝒮}({\hat{\theta }}_{1},{\hat{\theta }}_{2,1})=\frac{1_{Q_{𝒮}}}{K_{Q_{𝒮}}(\theta )}\frac{1}{\sqrt{{\left(2\pi \right)}^{K}\left|\sum_{{\hat{\theta }}_{1}}\right|}}\exp\left\{-\frac{1}{2}{\left({\hat{\theta }}_{1}-\theta \right)}^{\prime }P({\hat{\theta }}_{1}-\theta )\right\}\frac{1}{\sigma_{2,1}}\phi \left(\frac{{\hat{\theta }}_{2,1}-\theta_{1}}{\sigma_{2,1}}\right).$$

Let $p_{ij}$ (i,j=1,…,K) denote ${\mathrm{ij}}^{\text{th}}$ entry in P. Expanding the terms in the exponents and re‐arranging we get

(11) $$\begin{array}{ll} f_{𝒮}({\hat{\theta }}_{1},{\hat{\theta }}_{2,1}) & =\frac{1_{Q_{𝒮}}}{K_{Q_{𝒮}}(\theta )}\frac{1}{\sqrt{{\left(2\pi \right)}^{K}\left|\sum_{{\hat{\theta }}_{1}}\right|}}\frac{1}{\sqrt{2\pi \sigma_{2,1}^{2}}}\exp\left\{\frac{1}{2}\left[2\left(\overset{K}{\underset{i=1}{\sum }}\;p_{ij}{\hat{\theta }}_{1,i}+\frac{1}{\sigma_{2,1}^{2}}{\hat{\theta }}_{2,1}\right)\theta_{1}+2\overset{K}{\underset{i=2}{\sum }}\left(\overset{K}{\underset{j=1}{\sum }}\;p_{ij}{\hat{\theta }}_{1,j}\right)\theta_{i}\right]\right\}\\ & \;\times \exp\left\{-\frac{1}{2}\left(\theta^{\prime }P\;\theta +\frac{1}{\sigma_{2,1}^{2}}\theta_{1}^{2}+{\hat{\theta }}_{1}^{\prime }P\;{\hat{\theta }}_{1}+\frac{1}{\sigma_{2,1}^{2}}{\hat{\theta }}_{2,1}^{2}\right)\right\}. \end{array}$$

Define $T_{1}=\sum_{i=1}^{K}p_{1i}{\hat{\theta }}_{1,i}+\frac{1}{\sigma_{2,1}^{2}}{\hat{\theta }}_{2,1}$ and $T_{i}=\sum_{j=1}^{K}p_{ij}{\hat{\theta }}_{1,j}$ (i=2,…,K). The joint density $f_{𝒮}({\hat{\theta }}_{1},{\hat{\theta }}_{2,1})$ given by expression (11) has the exponential family form so that the vector ${\left(T_{1},\ldots ,T_{K}\right)}^{\prime }$ is sufficient and complete statistic while estimating $\theta_{1}$.

A linear transformation of ${\left(T_{1},\ldots ,T_{K}\right)}^{\prime }$ is also sufficient and complete statistic while estimating $\theta_{1}$. Consequently, from the simplification of the expression $T_{1}+\sum_{j=2}^{K}(q_{ij}/q_{1i})T_{j}$ (i=1,…,K), $\left({\hat{\theta }}_{1}^{*},\ldots ,{\hat{\theta }}_{K}^{*}\right)$, where ${\hat{\theta }}_{i}^{*}={\hat{\theta }}_{1,i}+\frac{q_{1i}}{\sigma_{2,1}^{2}}{\hat{\theta }}_{2,1}$, is sufficient and complete statistic while estimating $\theta_{1}$. Conditional on $Q_{𝒮}$, the UMVCUE is the expression for $E[{\hat{\theta }}_{2,1}|{\hat{\theta }}_{1}^{*},\ldots ,{\hat{\theta }}_{K}^{*}]$ and so we seek the density

$$f_{Q_{𝒮}}({\hat{\theta }}_{2,1}|{\hat{\theta }}_{1}^{*},\ldots ,{\hat{\theta }}_{K}^{*})=\frac{f_{Q_{S}}({\hat{\theta }}_{2,1},{\hat{\theta }}_{1}^{*},\ldots ,{\hat{\theta }}_{K}^{*})}{f_{Q_{𝒮}}({\hat{\theta }}_{1}^{*},\ldots ,{\hat{\theta }}_{K}^{*})}.$$

Let g(x) denote the density of a multivariate normal distribution for the random vector x. Further, let $\theta^{*}$ and $q_{1}$ denote the vectors ${\left({\hat{\theta }}_{1}^{*},\ldots ,{\hat{\theta }}_{K}^{*}\right)}^{\prime }$ and ${\left(q_{11},\ldots ,q_{1K}\right)}^{\prime }$, respectively. Then $f_{Q_{𝒮}}({\hat{\theta }}_{2,1},{\hat{\theta }}_{1}^{*},\ldots ,{\hat{\theta }}_{K}^{*})$ is given by

$$\frac{1_{Q_{𝒮}}}{K_{Q_{𝒮}}(\theta )}g\left(\theta^{*}-\frac{{\hat{\theta }}_{2,1}}{\sigma_{2,1}^{2}}q_{1}\right)\frac{1}{\sigma_{2,1}}\phi \left(\frac{{\hat{\theta }}_{2,1}-\theta_{1}}{\sigma_{2,1}}\right).$$

The density $f_{Q_{𝒮}}({\hat{\theta }}_{1}^{*},\ldots ,{\hat{\theta }}_{K}^{*})$ is obtained by integrating out ${\hat{\theta }}_{2,1}$ in the density $f_{Q_{𝒮}}({\hat{\theta }}_{2,1},{\hat{\theta }}_{1}^{*},\ldots ,{\hat{\theta }}_{K}^{*})$ so that

$$f_{Q_{𝒮}}({\hat{\theta }}_{2,1}|{\hat{\theta }}_{1}^{*},\ldots ,{\hat{\theta }}_{K}^{*})=\frac{g\left(\theta^{*}-\frac{{\hat{\theta }}_{2,1}}{\sigma_{2,1}^{2}}q_{1}\right)\frac{1}{\sigma_{2,1}}\phi \left(\frac{{\hat{\theta }}_{2,1}-\theta_{1}}{\sigma_{2,1}}\right)}{\int_{B_{L}}^{B_{U}}g\left(\theta^{*}-\frac{{\hat{\theta }}_{2,1}}{\sigma_{2,1}^{2}}q_{1}\right)\frac{1}{\sigma_{2,1}}\phi \left(\frac{{\hat{\theta }}_{2,1}-\theta_{1}}{\sigma_{2,1}}\right)d{\hat{\theta }}_{2,1}}$$

The UMVCUE is obtained by getting the expected value of the unbiased ${\hat{\theta }}_{2,1}$ by solving

$$E[{\hat{\theta }}_{2,1}|{\hat{\theta }}_{1}^{*},\ldots ,{\hat{\theta }}_{K}^{*}]=\frac{\int_{B_{L}}^{B_{U}}{\hat{\theta }}_{2,1}g\left(\theta^{*}-\frac{{\hat{\theta }}_{2,1}}{\sigma_{2,1}^{2}}q_{1}\right)\frac{1}{\sigma_{2,1}}\phi \left(\frac{{\hat{\theta }}_{2,1}-\theta_{1}}{\sigma_{2,1}}\right)d{\hat{\theta }}_{2,1}}{\int_{B_{L}}^{B_{U}}g\left(\theta^{*}-\frac{{\hat{\theta }}_{2,1}}{\sigma_{2,1}^{2}}q_{1}\right)\frac{1}{\sigma_{2,1}}\phi \left(\frac{{\hat{\theta }}_{2,1}-\theta_{1}}{\sigma_{2,1}}\right)d{\hat{\theta }}_{2,1}}=\frac{\int_{B_{L}}^{B_{U}}\frac{{\hat{\theta }}_{2,1}}{\eta_{1}}\phi \left(\left({\hat{\theta }}_{2,1}-\frac{\sigma_{2,1}^{2}}{\sigma_{1,1}^{2}+\sigma_{2,1}^{2}}{\hat{\theta }}_{1}^{*}\right)/\eta_{1}\right)d{\hat{\theta }}_{2,1}}{\int_{B_{L}}^{B_{U}}\frac{1}{\eta_{1}}\phi \left(\left({\hat{\theta }}_{2,1}-\frac{\sigma_{2,1}^{2}}{\sigma_{1,1}^{2}+\sigma_{2,1}^{2}}{\hat{\theta }}_{1}^{*}\right)/\eta_{1}\right)d{\hat{\theta }}_{2,1}},$$

where the simplification to the expressions with $\eta_{1}$ follows from Robertson et al. The expression $\frac{\sigma_{2,1}^{2}}{\sigma_{1,1}^{2}+\sigma_{2,1}^{2}}{\hat{\theta }}_{1}^{*}$ simplifies to ${\hat{\theta }}_{1}$. Solving the intergral in the denominator is straightforward giving $\Phi \left\{\left(B_{U}-{\hat{\theta }}_{1}\right)/\eta_{1}\right\}-\Phi \left\{\left(B_{L}-{\hat{\theta }}_{1}\right)\eta_{1}\right\}$. The solution to the integral in the numerator has also been given by several authors including Kan and Robotti^30^ which gives

$${\hat{\theta }}_{1}\left(\Phi \left\{\left(B_{U}-{\hat{\theta }}_{1}\right)/\eta_{1}\right\}-\Phi \left\{\left(B_{L}-{\hat{\theta }}_{1}\right)/\eta_{1}\right\}\right)-\eta_{1}\left(\phi \left\{\left(B_{U}-{\hat{\theta }}_{1}\right)/\eta_{1}\right\}-\phi \left\{\left(B_{L}-{\hat{\theta }}_{1}\right)/\eta_{1}\right\}\right).$$

Dividing the expressions for the numerator and denominator, expression (10) gives the UMVCUE for $\theta_{j}$ when 𝒮={j}. The UMVCUE is given by the same expression if more than one treatment continues to stage 2. This is because stage 2 estimates are not used for selection so that the terms related to them cancel out in the derivation.

### 4.3 UMVCUEs for the selection rules in Table 1

To get specific UMVCUEs corresponding to the selection rules in Table 1, we have derived expressions for $B_{L}$ and $B_{U}$ in the Supplementary Material and substituted in the general form of the UMVCUE given by expression (10). For the case of an experimental treatment continuing to stage 2 based on its comparison with the control and not the results from the other experimental treatments, that is, the first and the second selection rules in Table 1, for j∈𝒮, the UMVCUE for $\theta_{j}$ is given by

(12) $${\hat{\theta }}_{j,UMV}={\hat{\theta }}_{j}+\frac{\sigma_{2,j}^{2}}{\sqrt{\sigma_{1,j}^{2}+\sigma_{2,j}^{2}}}\frac{\phi \left(g(W_{j})\right)}{1-\Phi \left(g(W_{j})\right)},$$

where $W_{j}$ is the upper bound described in Section 2.5, and $g(W_{j})=\left(\sqrt{\sigma_{1,j}^{2}+\sigma_{2,j}^{2}}/\sigma_{1,j}^{2}\right)\left({\hat{\theta }}_{j}-W_{j}\right)$. For the third and fourth selection rules in Table 1, let l be the index for the treatment that corresponds to $W_{j}$. For example, in Row 3 (third selection rule), if $W_{j}={\hat{\theta }}_{1,3}$, l=3. For the third and fourth selection rules in Table 1, for j∈𝒮,

(13) $${\hat{\theta }}_{j,UMV}={\hat{\theta }}_{j}-\frac{\sigma_{2,j}^{2}}{\sqrt{\sigma_{1,j}^{2}+\sigma_{2,j}^{2}}}\frac{\phi \left(A_{1}\right)-\phi \left(A_{2}\right)}{\Phi \left(A_{1}\right)-\Phi \left(A_{2}\right)},$$

where expressions for $A_{1}$ and $A_{2}$ are given in Table 2. For the fifth selection rule in Table 1, if $W_{j}=b$, expression (12) is used to compute the estimate while if $W_{j}\neq b$, expression (13) is used with expressions for $A_{1}$ and $A_{2}$ being those of the third selection rule (Row 1 in Table 2). Computation for estimates corresponding to the sixth selection rule is similar with expressions for $A_{1}$ and $A_{2}$ being those of the fourth selection rule (Row 2 in Table 2).

**TABLE 2:** Expressions for $A_{1}$ and $A_{2}$ in expression (13)

	$q_{jl}>q(𝒮)$ ^a^		$q_{jl}<q(𝒮)$ ^a^	
Selected experimental treatment	$A_{1}$	$A_{2}$	$A_{1}$	$A_{2}$
Treatment with smallest log HR	$\frac{\left(\sigma_{2,j}^{2}+q_{jk}\right){\hat{\theta }}_{j}-\left(\sigma_{2,j}^{2}W_{j}+q_{jk}{\hat{\theta }}_{2,j}\right)}{\left(\sigma_{2,j}^{2}/\sqrt{\sigma_{1,j}^{2}+\sigma_{2,j}^{2}}\right)\left(\sigma_{1,j}^{2}-q_{jk}\right)}$	−∞	∞	$\frac{\left(\sigma_{2,j}^{2}+q_{jk}\right){\hat{\theta }}_{j}-\left(\sigma_{2,j}^{2}W_{j}+q_{jk}{\hat{\theta }}_{2,j}\right)}{\left(\sigma_{2,j}^{2}/\sqrt{\sigma_{1,j}^{2}+\sigma_{2,j}^{2}}\right)\left(\sigma_{1,j}^{2}-q_{jk}\right)}$
Treatment with smallest P‐value	$\frac{\left(\sigma_{2,j}^{2}\sigma_{1,k}+\sigma_{1,j}q_{jk}\right){\hat{\theta }}_{j}-\left(\sigma_{2,j}^{2}\sigma_{1,k}W_{j}+\sigma_{1,j}q_{jk}{\hat{\theta }}_{2,j}\right)}{\left(\sigma_{2,j}^{2}/\sqrt{\sigma_{1,j}^{2}+\sigma_{2,j}^{2}}\right)\left(\sigma_{1,j}^{2}\sigma_{1,k}-\sigma_{1,j}q_{jk}\right)}$	−∞	∞	$\frac{\left(\sigma_{2,j}^{2}\sigma_{1,k}+\sigma_{1,j}q_{jk}\right){\hat{\theta }}_{j}-\left(\sigma_{2,j}^{2}\sigma_{1,k}W_{j}+\sigma_{1,j}q_{jk}{\hat{\theta }}_{2,j}\right)}{\left(\sigma_{2,j}^{2}/\sqrt{\sigma_{1,j}^{2}+\sigma_{2,j}^{2}}\right)\left(\sigma_{1,j}^{2}\sigma_{1,k}-\sigma_{1,j}q_{jk}\right)}$

a For selecting the treatment with the smallest log HR, $q(𝒮)=\sigma_{1,j}^{2}$ while for the other selection rule $q(𝒮)=\sigma_{1,j}\sigma_{1,l}$.

## 5 EXAMPLE

To demonstrate how to compute estimates in Sections 2.6, 3, and 4, we construct a two‐stage adaptive trial from the single stage double blind CANMAT trial.^37^ The CANMAT trial recruited patients with bipolar I disorder who had a recent acute mood (manic or depressive) episode. The aim of the CANMAT trial was to determine optimal duration of taking adjunct atypical antipsychotic. Patients were randomised to mood stabilizer plus either two (Control), 24 (“Treatment 1”) or 52 (“Treatment 2”) weeks of atypical antipsychotic. The primary outcome is time to relapse to any mood episode.

The CANMAT trial had severe recruitment challenges and if this is typical for trials in this disease, clinical trials in this condition could benefit from two‐stage adaptive designs because, for example, it offers opportunity to stop early for futility. The CANMAT trial recruited from 17 sites in Canada and Brazil. The target sample size was 180 patients per group however the trial stopped after a total of 159 patients (52, 54, and 53 in control and treatments 1 and 2, respectively) because of slower recruitment than anticipated and funding expiry.^37^ We extracted data points from the CANMAT trial Kaplan‐Meier plot using the Webplotdigitizer^38^ and reconstructed the survival times using the method of Guyot et al.^39^ From Table 3, HR estimates from the reconstructed data are similar to those from the original data. The slight difference can be attributed partly to adjusting for potential confounders in the CANMAT trial (original) analysis.

**TABLE 3:** Worked example estimates based on Cox's proportional hazards model

	Cox's HR estimates		Reconstructed data^a^							
	Original	Reconstructed	Unadjusted quantities				Bias adjusted estimates			
	data	data	${\hat{\theta }}_{1,j}$	$V_{1,j}$	${\hat{\theta }}_{j}$	$V_{j}$	${\hat{\theta }}_{j,UMV}$	${\hat{\theta }}_{j,SI}$	${\hat{\theta }}_{j,MI}$	${\hat{\theta }}_{j,SH}$
Treatment 1	0.53	0.51	−0.5284	8.0705	− **0.6528**	16.6260	−0.6146	−0.5922	−0.5744	−0.6754
Treatment 2	0.63	0.56	−0.5327	8.7239	− **0.5796**	16.7495	−0.5281	−0.5110	−0.4890	−0.6057

Abbreviation: HR, Hazard ratio.

a j=1 and j=2 for treatments 1 and 2, respectively.

In the constructed phase II/III clinical trial, we assume uniform recruitment and since the CANMAT trial had recruitment difficulties, that this is over three years. We assume 1:1:1 random allocation for the control, treatments 1 and 2 within blocks of size three. Allocation of patients from each treatment, for example 52 patients for control, to different blocks was random with equal probability.

**FIGURE 1:**
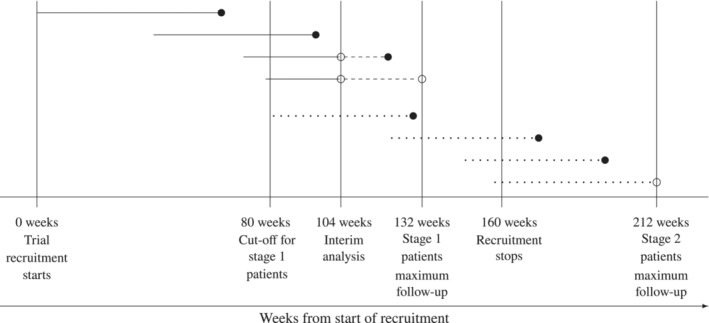
Constructed two‐stage trial based on CANMAT trial. Vertical lines correspond to key timepoints in the design. A horizontal line ending with a circle corresponds to a patient with the left hand side edge being parallel to time of recruitmentment. A filled circle corresponds to time of relapse whilst a nonfilled circle indicates that a relapse has not yet occurred at that key timepoint in the design. Lines with a continuous segment correspond to stage 1 patients whilst dotted lines correspond to stage 2 patients

Figure 1 gives the key aspects of the constructed two‐stage trial. Considering that the CANMAT trial maximum patient follow up was 52 weeks, the recruitment was challenging, most patients relapse within 6 months^37^ and limited funding and hence trial duration, we assume interim analysis based on calendar time with stage 1 patients consisting of those recruited in the first 80 weeks (approximately 1.5 years). Since the difference between treatments 1 and 2 is the duration of taking adjunct atypical antipsychotic (24 vs 52 weeks), we assume interim analysis of stage 1 patients with data collected until 104 weeks from start of recruitment so that patients included in the interim analysis have follow‐up of at least 24 weeks. This was informed by the fact that most patients relapse within 6 months. Between weeks 80 and 104 weeks, the trial continues recruitment and random allocation to the three groups. Patients recruited in this period and after 104 weeks form stage 2 patients. Note that since it is 24 weeks between recruiting the last stage 1 patient and performing interim analysis, if only one experimental treatment is selected to continue to stage 2, patients randomised to the dropped experimental treatment in stage 2 can be switched to the selected treatment group.

For the follow up in stage 2 of stage 1 patients who have not relapsed at the interim analysis, from recruitment, the natural choice is to follow them for 52 weeks which was the maximum follow up of patients in the CANMAT trial.^40^ Maximum follow‐up is 52 weeks after the last recruitment since as in the CANMAT trial, we assume a patient is followed for up to 52 weeks. Longer use of atypical antipsychotics is associated with more side effects.^37^ Therefore, we use a selection rule where all treatments that are promising based on efficacy continue to stage 2 so that sufficient safety data are collected from apparently effective treatments. Specifically, a treatment continues to stage 2 if pairwise P‐value ≤0.2. This corresponds to the last selection rule in Table 1 with a=0.2.

The constructed phase II/III clinical trial data, their format and the R code used to compute various estimates are in the repository “https://github.com/KimaniPK/Treatment‐Selection‐with‐TTE‐data”. In this section, we give estimates based on the Cox's proportional hazard model. Estimates based on the logrank score statistic are similar and are presented in Section 11.1 in the Supplementary Material. The unadjusted quantities from observed data that are needed to compute various bias adjusted estimates are log HR estimates and Fisher's information obtained from interim (stage 1) analysis and naive analysis. These are given in Table 3. From stage 1 log HRs and Fisher's information, stage 1 P‐values can be computed and both are less than 0.2 so that the trial continues to stage 2 with treatments 1 and 2 and with a=0.2, $W_{1}=-0.2948$ and $W_{2}=-0.2849$.

Bias adjusted estimates are given in Table 3. From expressions for variances for estimated log HRs in Section 2.1, and naive estimator expression (2) and UMVCUE expression (12) that corresponds to the selection rule used in this example, the unbiased estimates (UMVCUEs) can be computed. This gives the unbiased log HR estimates for effects of treatments 1 and 2 as ${\hat{\theta }}_{1,UMV}=-0.6146$ and ${\hat{\theta }}_{2,UMV}=-0.5281$, respectively.

Covariance of stage 1 log hazard ratios estimates is 0.0522 (correlation is 0.4377). The joint density of stage 1 treatments 1 and 2 log HR estimates, the expression for probability of continuing to stage 2 with both treatments and the expressions for the expected values for the two log HRs are given in the Supplementary Material. Using those expressions and expressions (2), (3) and (8) gives single iteration bias subtracted estimates for effects of treatments 1 and 2 as ${\hat{\theta }}_{1,SI}=-0.5922$ and ${\hat{\theta }}_{2,SI}=-0.5110$, respectively. Computation of multiple iterations bias subtracted estimates is similar, with the only difference being the inclusion of an iteration step because of using expression (9) rather than expression (8). The multiple iterations bias subtracted estimates for effects of treatments 1 and 2 are ${\hat{\theta }}_{1,MI}=-0.5744$ and ${\hat{\theta }}_{2,MI}=-0.4890$, respectively.

For the shrinkage estimator, we take ω=0.5. This is informed by uniform recruitment and stage 1 patients being those recruited until the 50% point in the trial. The function to estimate $\nu^{2}$ and stage 1 treatments 1 and 2 shrinkage estimates is in the Supplementary Material. We obtain $\nu^{2}=0$ and ${\hat{\theta }}_{1,SH}^{\left(2\right)}={\hat{\theta }}_{2,SH}^{\left(2\right)}=$‐0.5809 and consequently, treatments 1 and 2 two‐stage shrinkage estimates are ${\hat{\theta }}_{1,SH}=-0.6754$ and ${\hat{\theta }}_{2,SH}=-0.6057$, respectively.

The shrinkage estimates show slightly bigger treatment effects than the naive estimates (in Table 3, the naive estimates are bolded). The other bias adjusted estimates exhibit smaller effects than the naive estimates. As it would be expected in most datasets, multiple iterations bias subtracted estimates correct for biases more than the single iteration bias subtracted estimates.

## 6 COMPARISON OF THE ESTIMATORS USING A SIMULATION STUDY

### 6.1 Simulation study scenarios

We use simulations to compare the properties of the new UMVCUE, the naive estimator and the existing bias adjusted estimators by Brückner et al that are described in Section 3. In the simulations, we assume proportional Weibull hazard functions. The hazard function corresponding to the control treatment is given by $h_{0}(t)=\lambda \gamma t^{\gamma -1}$, where t is time and λ and γ are the scale and shape parameters, respectively. The hazard function corresponding to experimental treatment j is $h_{j}(t)=e^{\theta_{j}}h_{0}(t)$. In all simulations, we will take γ=0.5. We do not expect different values for γ to impact the properties of the estimators.^19^

The simulation study is based on the fifth selection rule in Table 1, that is, selecting the treatment that has the smallest stage 1 log hazard ratio that is below a futility value b. In all cases, the futility boundary b=0 so that a treatment is selected if it has the smallest observed log HR that is less than 0. The properties of the different estimators depend on several aspects such as the number of experimental treatments and information fraction of stage 1. These have been explored extensively by several authors.^12^, ^15^, ^36^ Specific to time to event data is the recruitment rate and how long patients without events in stage 1 are followed in stage 2. These aspects have been explored and are not expected to have impact on the estimators.^19^ Therefore, in the main paper we consider the case of K=4 but with different configurations for the true vector ${\left(\theta_{1},\ldots ,\theta_{4}\right)}^{\prime }$.

Sample sizes in simulations are such that, if it was a single‐stage trial and the true hazard ratio (HR) is 0.8, the combined stage 1 and stage 2 number of deaths from the control and the selected experimental treatment correspond to power of 80%. For 80% power, 632 deaths (rounded up to an even number) are required. We fixed deaths from patients recruited in stage 2, that is stage 2 patients, to be 316.

Recalling that we take the shape parameter γ=0.5 and taking time t in the hazard function to be in days, we set the scale parameter for the control treatment such that the median is 365 days, that is, $\lambda_{0}=\ln(2)/365$. We determined the number of deaths in stage 1 assuming the four HRs corresponding to the four experimental treatments are all equal to 0.8, that is, $\lambda_{i}=0.8\lambda_{0}$ (i=1,…,4). The scale parameter for the experimental treatment can be computed from the HR and the scale parameter for the control. Assuming that on average 1 person is recruited per day in stage 1, we set the interim analysis to be performed after 717 deaths which, after rounding off, corresponds to the expected number of events comparing each experimental treatment to the control to be 298 (159 in control and 139 in the experimental treatments).

Assuming a uniform recruitment rate of 1 patient per day in stage 2, for the assumed hazard functions when HR is 0.8, we expect 316 events from stage 2 patients to be observed after 2 years. Therefore we fixed to follow the stage 1 patients from the control and selected experimental treatment without events at the interim analysis to be followed for 1 year post interim analysis.

For each scenario, we simulated 100 000 trials and for each trial, we computed the naive estimate and four bias ajusted estimates corresponding to estimators in Sections 3 and 4. We assessed the properties of the estimates corresponding to the five estimators using bias and root mean squared error (RMSE). We computed the biases and RMSEs conditional on the selected treatment. For example, let ${\hat{\theta }}_{N_{j}}^{\left(i\right)}$ denote the naive estimate for the $i^{\text{th}}$ simulated trial and $1_{\left[j=l\right]}^{\left(i\right)}$ the indicator that j=l in the $i^{\text{th}}$ simulation trial. Then, for example for the case of selecting treatment three, that is j=3, bias and RMSE for the naive estimator was computed as

$$\text{Bias}=\frac{\sum_{i=1}^{100,000}({\hat{\theta }}_{j}^{\left(i\right)}-\theta_{j})1_{\left[j=3\right]}^{\left(i\right)}}{\sum_{i=1}^{10,000}1_{\left[j=3\right]}^{\left(i\right)}}\;\text{and}\;\text{RMSE}=\sqrt{\frac{\sum_{i=1}^{100,000}{\left({\hat{\theta }}_{j}^{\left(i\right)}-\theta_{j}\right)}^{2}1_{\left[j=3\right]}^{\left(i\right)}}{\sum_{i=1}^{10,000}1_{\left[j=3\right]}^{\left(i\right)}}}.$$

Note that these biases and RMSEs are conditional on continuing to stage 2 and hence not stopping for futility. Estimators in Sections 3.1 and 4 adjust for both treatment selection and possibility of stopping for futility explicitly.

### 6.2 Results

In this section, we give results when estimation is based on Cox's proportional hazards model. In a small proportion of simulated trials, there was no convergence while computing ${\hat{\theta }}_{j,MI}$ estimate. This information and the number of events from simulated trials are summarized in the Supplementary Material. Simulated properties for the various estimators are summarized in Table 4. Column 1 indicates the treatment for which results in a row corresponds to while column 2 gives the simulated probability of selecting the treatment in column 1. The probabilities for the four treatments do not add up to one because the trial does not continue to stage 2 if all four observed log HRs are below 0. Columns 3 to 7 give biases for the five estimators while columns 8 to 12 give their RMSEs computed as described in Section 6.1. Biases and RMSEs for ${\hat{\theta }}_{j,MI}$ do not include simulated trials where there is no convergence. Since in all scenarios the four true log hazard ratios are less or equal to zero and a negative log hazard ratio indicates an experimental treatment is more effective than the control, biases with negative and positive signs in Table 4 indicate overestimating and underestimating treatment effects, respectively.

**TABLE 4:** Simulated biases and root mean squared errors for the various estimators for the log hazard ratios

	Selection	Simulated bias^a^					Root mean squared error^b^				
Treatment	probability	${\hat{\theta }}_{j}$	${\hat{\theta }}_{j,UMV}$	${\hat{\theta }}_{j,MI}$	${\hat{\theta }}_{j,SI}$	${\hat{\theta }}_{j,SH}$	${\hat{\theta }}_{j}$	${\hat{\theta }}_{j,UMV}$	${\hat{\theta }}_{j,MI}$	${\hat{\theta }}_{j,SI}$	${\hat{\theta }}_{j,SH}$
Scenario 1: True log hazard ratios are $\theta_{1}=\theta_{2}=\theta_{3}=\theta_{4}=0$											
1	0.1998	−0.0526	− **0.0022**	0.0169	−0.0091	−0.0331	0.0864	0.0936	0.0942	0.0830	**0.0735**
2	0.2022	−0.0530	− **0.0033**	0.0156	−0.0098	−0.0348	0.0861	0.0927	0.0927	0.0822	**0.0738**
3	0.1974	−0.0536	− **0.0037**	0.0155	−0.0104	−0.0281	0.0870	0.0933	0.0936	0.0830	**0.0696**
4	0.1994	−0.0524	− **0.0021**	0.0170	−0.0089	−0.0342	0.0861	0.0932	0.0936	0.0826	**0.0738**
Scenario 2: True log hazard ratios are $\theta_{1}=\theta_{2}=\theta_{3}=\theta_{4}=-0.2231$											
1	0.2463	−0.0389	**0.0002**	0.0164	−0.0087	−0.0175	0.0825	0.0893	0.0904	0.0794	**0.0728**
2	0.2504	−0.0390	**0.0006**	0.0167	−0.0087	−0.0189	0.0826	0.0898	0.0908	0.0796	**0.0734**
3	0.2518	−0.0402	− **0.0013**	0.0149	−0.0101	−0.0023	0.0826	0.0889	0.0896	0.0790	**0.0715**
4	0.2507	−0.0397	− **0.0002**	0.0159	−0.0094	−0.0195	0.0833	0.0899	0.0910	0.0801	**0.0740**
Scenario 3: True log hazard ratios are $\theta_{1}=0$, $\theta_{2}=-0.1393$, $\theta_{3}=-0.3011$ and $\theta_{4}=-0.5108$											
2	0.0009	−0.1017	−0.0174	− **0.0075**	−0.0580	−0.0950	0.1243	0.0945	**0.0914**	0.0962	0.1190
3	0.0503	−0.0563	**0.0001**	0.0075	−0.0286	−0.0340	0.0933	0.0925	**0.0911**	0.0834	0.0838
4	0.9488	−0.0034	− **0.0002**	0.0055	0.0023	0.0182	**0.0793**	0.0818	0.0841	0.0816	0.0821
Scenario 4: True log hazard ratios are $\theta_{1}=\theta_{2}=\theta_{3}=-0.9163$ and $\theta_{4}=-1.0498$											
1	0.1041	−0.0624	− **0.0025**	0.0143	−0.0240	−0.0377	0.0961	0.0963	0.0949	0.0835	**0.0806**
2	0.1038	−0.0610	− **0.0012**	0.0156	−0.0226	−0.0378	0.0946	0.0944	0.0938	0.0822	**0.0804**
3	0.1040	−0.0627	− **0.0037**	0.0134	−0.0245	−0.0456	0.0959	0.0948	0.0938	**0.0829**	0.0865
4	0.6882	−0.0203	−0.0012	0.0150	**0.0002**	0.0073	**0.0794**	0.0893	0.0927	0.0829	0.0765

a Smallest bias in bold.

b Smallest Root mean squared error in bold.

**FIGURE 2:**
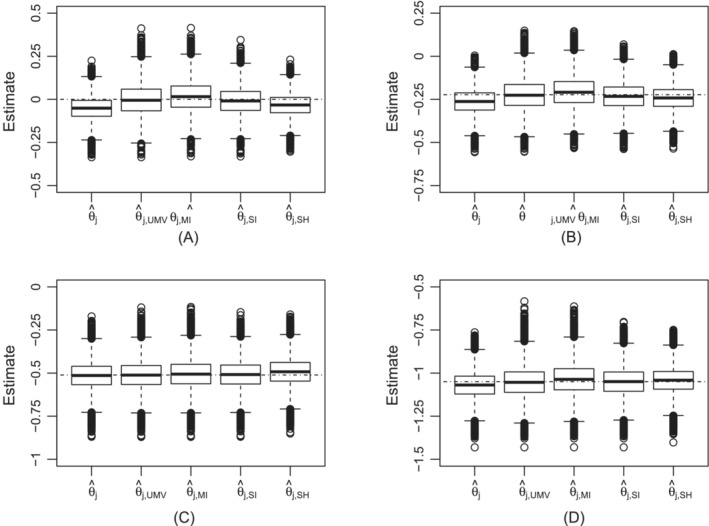
Boxplots for treatment four log hazard ratio estimates for (A‐D) scenarios 1 to 4. The true log hazard ratios are represented by the dashed and dotted lines

Four scenarios based on different values for the true log HRs vector ${\left(\theta_{1},\theta_{2},\theta_{3},\theta_{4}\right)}^{\prime }$ are presented. In the first scenario ($\theta_{1}=\theta_{2}=\theta_{3}=\theta_{4}=0$), all treatments including the control have equal hazards of death so that a treatment being considered best is by chance and when the decision is to continue to stage 2, the observed log HR is either equal to the true value or overestimates log HR. For this scenario, the probability of continuing to stage 2 is 0.80 with, as expected because of equal true log HRs, approximately equal simulated probabilities for selecting each of the four experimental treatments. Also, biases and RMSEs for the four treatments are approximately equal. As expected, the naive estimator (${\hat{\theta }}_{j}$) overestimates treatments' effects. The approximate UMVCUE (${\hat{\theta }}_{j,UMV}$) has the smallest biases among all the estimators, being practically unbiased. The multiple iterations bias subtracted estimator (${\hat{\theta }}_{j,MI}$) slightly underestimates treatments' effects. The RMSEs for ${\hat{\theta }}_{j,UMV}$ and ${\hat{\theta }}_{j,MI}$ are approximately equal but bigger than those for the naive estimator. The single iteration bias subtracted estimator (${\hat{\theta }}_{j,SI}$) slightly overestimates log HR, that is, it undercorrects for bias. It is better than the naive estimator since its RMSEs are also smaller than those for the naive estimator. For this scenario, ${\hat{\theta }}_{j,SI}$ is also better than the multiple iterations bias subtracted estimator since, albeit overestimating true log HR, both its biases and RMSEs are smaller. Making a decision on which estimator between the single iteration bias subtracted estimator (${\hat{\theta }}_{j,SI}$) and the UMVCUE (${\hat{\theta }}_{j,UMV}$) is better is not clear cut since ${\hat{\theta }}_{j,UMV}$ is practically unbiased whilst ${\hat{\theta }}_{j,SI}$ has markedly smaller RMSEs. The shrinkage estimator overestimates treatments' effects in this scenario and has the largest biases among the bias adjusted estimators. However, it has smallest RMSEs among all estimators. To enable clearer comparison of all the estimators, boxplots in Figure 2A complements properties for the effects corresponding to experimental treatment four in Table 4. Three quarters of the naive estimates provide bigger treatment effects than the true treatment effect. In comparison to the naive estimates, the shrinkage estimates show a small shift of estimates reducing mean bias but with almost three quarters of the estimates providing bigger treatment effects than the true effect. The spreads of the naive and shrinkage estimates are approximately equal. The UMVCUE is approximately symmetrically distributed around the true log HR. The multiple iterations bias subtracted estimator is also approximately symmetrically distributed around the true log HR although it slightly overcorrects for bias, exhibiting more bias than UMVCUE. Although they have wider spread than the naive and shrinkage estimates, their range above line of true log HR is equal to the range of naive estimates below line of the true log HR. Thus we would consider the UMVCUE and multiple iterations bias subtracted estimator better than both the naive and shrinkage estimators. If a slight overestimation is acceptable, for this scenario, the single iteration bias subtracted estimator performs best as it is approximately mean unbiased and has smaller RMSEs than the UMVCUE and the multiple iterations bias subtracted estimator.

For Scenario 2, $\theta_{1}=\theta_{2}=\theta_{3}=\theta_{4}=\ln(0.8)=-0.2231$ so that all experimental treatments are better than the control and equally effective. From Table 4, the probability of continuing to stage 2 is almost 1 so that bias in this setting is attributed to treatment selection and not to the possibility of stopping for futility. Since all experimental treatments have equal hazards of death, their simulated probabilities of continuing to stage 2 are approximately equal. For this scenario, the biases of the naive estimators corresponding to the four experimental treatments are smaller than those for scenario 1. Considering the results for this scenario in Table 4 and boxplots of treatment four estimates in Figure 2B, we notice that the UMVCUE has negligible biases and also the other estimators have smaller biases compared to Scenario 1. However, we still make the same conclusion as scenario 1 when all estimators are compared.

The third scenario considers a case where the four treatments have distinct hazards of death ($\theta_{1}=0$, $\theta_{2}=-0.1393$, $\theta_{3}=-0.3011$ and $\theta_{4}=-0.5108$). In the simulations, the probabilities of continuing to stage 2 with treatment 3 and treatment 4 are 0.050 and 0.949, respectively. Treatment 1 is not selected and treatment 2 has negligible probability of being selected. For treatment 3 and treatment 4 that have high numbers of simulated trials, the UMVCUE has neglible biases which are markedly smaller compared to the biases for the other estimators. When the superior treatment 4 is selected (fifth last row in Table 4 and Figure 2C), the shrinkage estimator is only slightly biased while the other estimators, including the naive estimator, are practically mean unbiased. The RMSEs for all the estimators are close, being smallest for the naive estimator followed by the single iteration bias subtracted estimator. In this case of selecting treatment 4 in scenario 3, the naive estimator performs best followed very closely by the UMVCUE but the other estimators also perform well. When treatment 3 is selected, the single iteration bias subtracted estimator reduces bias substantially but retains noticeable bias and does not perform much better than the shrinkage estimator. Thus, the single iteration bias subtracted estimator does not perform well when an inferior treatment is selected. The UMVCUE is practically mean unbiased and multiple iterations bias subtracted estimator is approximately mean unbiased and these two estimators have similar RMSEs which are below those of the naive estimator so that they have better properties than the naive estimator. In summary, we consider UMVCUE and multiple iterations bias subtracted estimator to perform better than the other estimators when treatment 3 is selected. This is clearer to observe in Figure S1C in the Supplementary Material.

Scenario 4 ($\theta_{1}=\theta_{2}=\theta_{3}=-0.9163$ and $\theta_{4}=-1.0498$) is a case where all treatments are highly effective with treatment 4 more effective than the other three treatments that have equal effects. In the simulations, treatment 4 is selected with probability 0.688 while the other treatments are selected with approximately equal probability of 0.104. Selecting treatment 4 with high probability leads to the naive estimator having small bias. The naive estimator and all the estimators that have adjusted for bias have approximately equal biases and not very different RMSEs (last row in Table 4 and boxplots in Figure 2D) so that they perform equally well. When treatment 1, treatment 2 or treatment 3 is selected, the single iteration bias subtracted estimator retains substantial bias so that, as in Scenario 3, it does not perform well when an inferior treatment is selected. However, the UMVCUE and the multiple iterations bias subtracted estimator perform well. This is clearer to observe in Figure S1D in the Supplementary Material.

### 6.3 Summary findings from the simulation study

From the simulations, the naive estimator has substantial bias in some scenarios. The approximate UMVCUE has the smallest biases, being practically unbiased. The multiple iterations bias subtracted estimator is slightly more biased than the UMVCUE, mostly overcorrecting for bias. Apart from that, the two estimators have similar properties. They are approximately unbiased but in most scenarios, they have higher RMSEs than the naive estimator. This is because, from the boxplots, their estimates are approximately symmetric around the true value whereas when the naive estimator is biased, more than half of its estimates are below the true value but its smallest log hazard ratio is approximately equal to smallest log hazard ratios for the UMVCUE and the multiple iterations bias subtracted estimator. Therefore, in clinical trials where unbiased estimators are required, we consider the UMVCUE and multiple iterations bias subtracted estimator to be better than the naive estimator, with the former slightly better. The single iteration bias subtracted estimator does not eradicate bias fully, retaining substantial bias if the selected treatment is not the most effective, but it has smaller RMSEs compared to the UMVCUE and multiple iterations bias subtracted estimator and so it may be preferable in clinical trials where some overestimation is acceptable. The shrinkage estimator does not eradicate much bias but it has smallest RMSEs in most scenarios.

Findings for the case of two experimental treatments in stage 1 are similar (Supplementary Material, Tables S5 and S6 and Figures S2 and S3) so that the results can be generalized to values of K that are of practical relevance. We also performed simulations for the case of four treatments in stage 1 and smaller number of deaths (Supplementary Material, Tables S7 and S8 and Figures S4 and S5). Compared to the results in Section 6.2, there are more cases for nonconvergence when computing the multiple iterations bias subtracted estimates, the biases of the naive estimator are larger and RMSEs for all the estimators are larger. However, the conclusion from comparing the various estimators is similar.

Simulations results when estimation is based on the logrank score statistic are summarized in the Supplementary Material (Tables S10 to S15 and Figures S6 to S11). For Scenario 1 ($\theta_{1}=\theta_{2}=\theta_{3}=\theta_{4}=0$) and Scenario 2 ($\theta_{1}=\theta_{2}=\theta_{3}=\theta_{4}=-0.2231$), results are similar to those where estimation is based on Cox's proportional hazards model. For Scenario 3 and 4, in some cases, all the estimators underestimate treatments' effects slightly (eg, Figure S8). This is of little practical relevance since the biases are small and the treatment is highly effective. Like Kimani et al,^19^ we attribute underestimation to the fact that the asymptotic normal distributions in Section 2 are based on using few terms of the Taylor's expansion and is more accurate for log hazard ratios close to zero.^20^ A bigger problem with estimation based on the logrank score statistic is that, in Scenario 4 where all treatments have HRs less than 0.4, the asymptotic distribution approximation did not work well especially when the selected treatment is not the most effective and when the sample size is small. Therefore, we do not recommend estimation based on logrank score statistic if HRs <0.4 are considered plausible in a clinical trial.

## 7 DISCUSSION

Phase II/III clinical trials are efficient for testing multiple experimental treatments. However, in simulations based on typical sample sizes in confirmatory trials, we observed that in some scenarios the naive estimator for log hazard ratio has substantial biases arising from adaptation performed at an interim analysis so that it is necessary to have bias adjusted estimators. Brückner et al^18^ have shown how to adjust for treatment selection by estimating bias and subtracting it from the naive estimate (bias subtracted estimators) or by using shrinkage estimators. Extending uniformly minimum variance conditional unbiased estimator (UMVCUE) for normally distributed outcomes^13^, ^14^, ^15^, ^16^, ^17^ to time to event data, we have derived an approximate UMVCUE for the log hazard ratio (log HR). While also adjusting for possibility of stopping for futility, we have compared the biases and the mean squared errors of the new UMVCUE to those of the naive estimator and existing estimators.

In most scenarios, all the bias adjusted estimators we have considered perform better than the naive estimator. The shrinkage estimator does not eradicate much bias but performs best in terms of mean squared error. Our recommendation is to use the shrinkage estimator if the primary aim is to have an estimator whose variability does not exceed that of the naive estimator. The interpretation of shrinkage estimates needs to include an acknowledgement that biases of the shrinkage estimator are only slightly less than those of the naive estimator.

The single iteration bias subtracted estimator does not eradicate bias fully, overestimating treatments' effects. From the simulation study, while emphasizing that in practice true treatments' effects are unknown, the overestimation is only noticeable if the experimental treatment selected is not the most desired treatment to continue to stage 2 but even in this case, this estimator is still much less biased compared to the naive estimator. Also, in most scenarios it has smaller mean squared errors than the naive estimator. Thus, the single iteration biased subtracted estimator is preferable if one is ready to accept slight overestimation. Because the bias reduction for this estimator depends on the true values for the log hazard ratios, before conducting a clinical trial, we recommend assessing its properties using a simulation study of plausible hazard functions.

Regulators recommend that the magnitude of the bias of a naive point estimator is understood, effort is made to control the bias and the point estimator to be used in the analysis is prespecified.^41^, ^42^ So, we recommend that trialists choose in advance of clinical trial conduct to use either the new UMVCUE or the multiple iterations bias subtracted estimator. The two estimators are approximately unbiased and have similar mean squared errors. The UMVCUE, however, has simpler expressions for computing estimates, is approximately unbiased by derivation and in simulations had the smallest biases among all the estimators we considered. Expressions (12) and (13) cover several selection rules, and where it is possible to determine limits of integration as demonstrated in Section 2.5 and Table 1, steps in the Supplementary Material used to obtain expressions (12) and (13) can be used to get UMVCUE expressions for more selection rules. The multiple iterations bias subtracted estimator requires derivation for the expressions for biases which may not be straightforward and also, sometimes, there is no convergence of the iteration procedure which from simulations, is worse with higher number of treatments, smaller sample size and selecting a treatment that is not best in terms of true log HR. The risk of convergence is also higher for estimation based on logrank score statistic than estimation based on Cox's proportional hazards model and particularly high when selecting a treatment that is not the best in terms of true log HR. If trialists prefer the multiple iterations bias subtracted estimator, as mitigation in case there is no convergence for the multiple iterations bias subtracted estimator, it can be prespecified that UMVCUE estimate will be used in case of no convergence with the former.

Brückner et al^18^ considered another shrinkage estimator. In our setting, if we had included the estimator in our work, we expect the same conclusion as that we have made from comparing the shrinkage estimator in this paper to the other estimators. This is because from Brückner et al simulations, the shrinkage estimator we have considered in this paper performed better in most scenarios than the shrinkage estimator we have not considered. Also, in Brückner et al simulations, the two shrinkage estimators did not eradicate bias fully in most scenarios.

In simulations, we have used a single treatment selection rule to compare the various estimators. For other selection rules, we expect the UMVCUE to have least biases as it is approximately unbiased by derivation. The shrinkage estimator is likely to have largest biases and least MSEs among the bias adjusted estimators since this has been observed for different selection rules when patient outcomes are normally distributed.^12^, ^15^, ^32^ We expect the single iteration and multiple iterations estimators to eradicate most biases but we recommend simulations to compare them to the UMVCUE when a different selection rule is to be used in a trial.

The UMVCUE we have derived is not fully unbiased. One reason for this is following stage 1 patients without events before the interim analysis in stage 2 so that the independent increment structure is an approximate assumption. Bias can be reduced by not following stage 1 patients in stage 2. However, this can lead to not including many events which increases RMSE. Therefore, we recommend using a design that follows stage 1 patients in stage 2 but has an approximate independent increment structure.

We have considered estimation based on Cox's proportional hazards model and on the log rank score statistic. Trial investigators would need to prespecify the model to use in advance. In general, we recommend estimation based on Cox's proportional hazards model since its estimates have better properties than those based on the logrank score statistic, it is possible to include covariates and using standard statistical packages, it is easier to obtain parameter values for the distribution given by expression (1). Estimates based on the score statistic are attractive as they would be expected to align to the logrank test which is commonly used to test hypotheses. However, from literature^19^ and as we have observed from simulations, properties of the point estimators can be undesirable when the true hazard ratios of the treatments are less than 0.4. Therefore, we do not recommend estimation based on logrank score statistic if investigators believe hazard ratios less than 0.4 are plausible.

We have not considered confidence intervals with appropriate coverage probabilities. To obtain confidence intervals that adjust for treatment selection, we suggest extending the work of Magirr et al^43^ as was done for the case of subpopulation selection with time to event data.^19^ However, confidence intervals obtained using this method can be noninformative sometimes, especially for the cases where mutiple treatments continue to stage 2.^19^, ^43^ Therefore, development of new methods for constructing confidence intervals is necessary.

We have provided a program written in R^29^ that was used to compute the estimates in Section 5 which can enhance adoption of the methods in practice. Developing statistical packages can improve adoption and is future work.

## Supporting information




**Appendix S1** Supporting Information

## Data Availability

The data and the code used to obtain the estimates in Section 5 are available in the repository “https://github.com/KimaniPK/Treatment‐Selection‐with‐TTE‐data”.^22^ The Supplementary Material provides the derivations and simulations results that are not presented in the main paper.
