# Supplementary material for: Adjusting for treatment selection in phase II/III clinical trials with time to event data
Source: Stat Med. 2022 Nov 23;42(2):146–63. doi: 10.1002/sim.9606 (PMC10098876; doi:10.1002/sim.9606)
Supplement: Supplementary file 1 — Appendix S1 Supporting Information [file SIM-42-146-s001.pdf]

Adjusting for treatment selection in phase II/III clinical trials with time to event data

Supplementary material

1 Bounds for Bowden and Glimm (2008) selection rule

Bowden and Glimm [1] rule involves selecting a pre-specified number of the best experimental treatments. Table 1 summarises Bowden and Glimm selection rule for the case of selecting the best two treatments. When a futility threshold is added to the Bowden and Glimm selection rule so that it is also required that the stage 1 log hazard ratio (HR) of a selected treatment is $\leq b$, Table 2 is the case of selecting the best two treatments that have stage 1 log HR $\leq b$. The limits are the same for rule of selecting the best three (or more) experimental treatments that have stage 1 log HR $\leq b$ but only two treatments have observed stage 1 log HR $\leq b$ and hence only selecting the two best treatments.

Table 1: Bowden and Glimm selection rule

	Limits for individual	Selected treatments bounds conditional on other effects	
Treatment	treatments' effects	Lower (L_j)	Upper (W_j)
$1^{\dagger}$	$-\infty < \hat{\theta}_{1,j} < \infty$	$-\infty$	$\min\left(\{\hat{\theta}_{1,1},...,\hat{\theta}_{1,K}\} \setminus \hat{\theta}_{1,j}\right)$
$2^{\dagger}$	$\hat{\theta}_{1,j} < \hat{\theta}_{2,j} \leq \infty$	$\hat{\theta}_{1,j}$	$\min\left(\{\hat{\theta}_{1,1},...,\hat{\theta}_{1,K}\} \setminus \{\hat{\theta}_{1,j},\hat{\theta}_{2,j}\}\right)$
3	$\hat{\theta}_{2,j} < \hat{\theta}_{3,j} \leq \infty$	NA	NA
·	·	NA	NA
K	$\hat{\theta}_{2,j} < \hat{\theta}_{K,j} \leq \infty$	NA	NA

† Selected treatments, that is, $j \in S$ ($j = 1,2$).

Table 2: Modified Bowden and Glimm selection rule

Treatment	Limits for individual treatments' effects	Selected treatments bounds conditional on other effects	
		Lower (L_j)	Upper (W_j)
$1^{\dagger}$	$-\infty < \hat{\theta}_{1,j} < b$	$-\infty$	$\min\left(\{b, \hat{\theta}_{1,1}, ..., \hat{\theta}_{1,K}\} \setminus \hat{\theta}_{1,j}\right)$
$2^{\dagger}$	$\hat{\theta}_{1,j} < \hat{\theta}_{2,j} \leq b$	$\hat{\theta}_{1,j}$	$\min\left(\{b, \hat{\theta}_{1,1}, ..., \hat{\theta}_{1,K}\} \setminus \{\hat{\theta}_{1,j}, \hat{\theta}_{2,j}\}\right)$
3	$b < \hat{\theta}_{3,j} \leq \infty$	NA	NA
·	·	NA	NA
K	$b < \hat{\theta}_{K,j} \leq \infty$	NA	NA

† Selected treatments, that is, $j \in S$ $(j = 1, 2)$.

2 Matrix A in Section 2.5 in the main paper

$$A = \begin{pmatrix} 1 & 0 & 0 & \cdot & \cdot & \cdot & 0 \\ -1 & 1 & 0 & \cdot & \cdot & \cdot & 0 \\ -1 & 0 & 1 & \cdot & \cdot & \cdot & 0 \\ \cdot & \cdot & \cdot & \cdot & & & \cdot \\ \cdot & \cdot & \cdot & & \cdot & & \cdot \\ \cdot & \cdot & \cdot & & & \cdot & \cdot \\ -1 & 0 & 0 & \cdot & \cdot & \cdot & 1 \end{pmatrix}$$

3 Description for c in expression (7) in Section 2.5 in the main paper

Here, we give the components of vector $\boldsymbol{c}$ in expression (7) in Section 2.5 in the main paper. Using similar notation as Kimani *et al.* [2], with reference to $\Sigma_{\hat{\delta}_1}$, let $\Sigma_{i,j}$ be the ij^{th} entry, $\Sigma_{-i,-j}$ be the resulting matrix after excluding row i and column j, $\Sigma_{-i,(j)}$ be the resulting column vector after excluding i^{th} element of the jth column, $\Sigma_{(i),-j}$ be the resulting row vector after excluding the jth element of the ith row, and define

$$\tilde{\Sigma}_i = \Sigma_{-i,-i} - \frac{1}{\Sigma_{i,i}} \Sigma_{-i,(i)} \Sigma_{(i),-i}.$$

Denote by $\hat{\delta}_1$ the vector $(\hat{\delta}_{1,1}, ..., \hat{\delta}_{1,K})'$. Let $\delta_{(j)}$ and $\hat{\delta}_{1,(j)}$ be the resulting vectors after excluding the jth elements in δ and $\hat{\delta}_1$, respectively. Define $\delta_1^* = \left(\frac{b-\delta_1}{\Sigma_{1,1}}\right)$, $\delta_j^* = \left(\frac{-\delta_j}{\Sigma_{j,j}}\right)$ $(j = 2, ..., K)$ and $\tilde{\delta}_{(j)} = \delta_{(j)} + \Sigma_{-j,(j)} \delta_j^*$. Then the first entry in $\boldsymbol{c}$ is given by

$$-\frac{1}{\sqrt{\Sigma_{1,1}}} \phi \left(\frac{b - \delta_1}{\sqrt{\Sigma_{1,1}}} \right) \int_0^{\infty} ... \int_0^{\infty} f(\hat{\delta}_{1,(1)}) d\hat{\delta}_{1,2}^* ... d\hat{\delta}_{1,K}^*$$

where $f(\hat{\delta}_{1,(1)})$ is the density of a $MVN(\tilde{\delta}_{(1)},\tilde{\Sigma}_1)$ and the jth ($j=2,...,K$) entry in $\boldsymbol{c}$ is given by

$$\frac{1}{\sqrt{\Sigma_{j,j}}}\phi\left(\frac{-\delta_j}{\sqrt{\Sigma_{j,j}}}\right)\int_{-\infty}^{b}\int_{0}^{\infty}\cdots\int_{0}^{\infty} f(\hat{\delta}_{1,(j)})d\hat{\delta}^*_{1,1}...d\hat{\delta}^*_{1,j-1}d\hat{\delta}^*_{1,j+1}...d\hat{\delta}^*_{1,K}$$

where $f(\hat{\delta}_{1,(j)})$ is the density of a $MVN(\tilde{\delta}_{(j)},\tilde{\Sigma}_j)$. Note that computing the values for these integrals can be done using standard statistical programs.

Treatment 1 which is selected.

4 Prob(S) and expected values for other selection rules in Section 2.4 in the main document

4.1 Selecting treatment with smallest p-value that is below a futility value

For the rule of selecting the treatment with the smallest p-value that is below a futility value a, without loss of generality, if treatment 1 is selected, then

$$\text{Prob}(S)=\int_{-\infty}^{\Phi^{-1}(a)/\sqrt{V_{1,1}}}\int_{\hat{\theta}_{1,1}\sqrt{V_{1,1}/V_{1,2}}}^{\infty}\cdots\int_{\hat{\theta}_{1,1}\sqrt{V_{1,1}/V_{1,j}}}^{\infty} f(\hat{\theta}_{1,1},...,\hat{\theta}_{1,K})d\hat{\theta}_{1,K}\cdots d\hat{\theta}_{1,2}d\hat{\theta}_{1,1}.$$

As before, for simpler computations, a linear transformation with $\hat{\delta}_{1,1}=\hat{\theta}_{1,1}$ and $\hat{\delta}_{1,j}=\hat{\theta}_{1,j}-\hat{\theta}_{1,1}(\sqrt{V_{1,1}/V_{1,j}})$ is used. Let A be a $K\times K$ matrix where the first element in column one is 1 while j^{th} element in column one is equal to $-\sqrt{V_{1,1}/V_{1,j}}$. For column i ($i=2,...,K$), the ith element is equal to 1 while the other elements are all equal to zero. That is,

$$A=\begin{pmatrix} 1 & 0 & 0 & \cdot & \cdot & \cdot & 0\\ -\sqrt{\frac{V_{1,1}}{V_{1,2}}} & 1 & 0 & \cdot & \cdot & \cdot & 0\\ -\sqrt{\frac{V_{1,1}}{V_{1,3}}} & 0 & 1 & \cdot & \cdot & \cdot & 0\\ \cdot & \cdot & \cdot & \cdot & & & \cdot\\ \cdot & \cdot & \cdot & & \cdot & & \cdot\\ \cdot & \cdot & \cdot & & & \cdot & \cdot\\ -\sqrt{\frac{V_{1,1}}{V_{1,K}}} & 0 & 0 & \cdot & \cdot & \cdot & 1 \end{pmatrix}$$

Let $\hat{\theta}_1$ and $\hat{\delta}_1$ denote the vectors $(\hat{\theta}_{1,1},...,\hat{\theta}_{1,K})'$ and $(\hat{\delta}_{1,1},...,\hat{\delta}_{1,K})'$, respectively, where $\hat{\delta}_1=A\hat{\theta}_1$ so that $\hat{\delta}_{1,1}=\hat{\theta}_{1,1}$ and $\hat{\delta}_{1,j}=\hat{\theta}_{1,j}-\hat{\theta}_{1,1}(\sqrt{V_{1,1}/V_{1,j}})$ ($j=2,...,K$). Setting $b=\Phi^{-1}(a)/\sqrt{V_{1,1}}$, Prob$(S)$ and $E\left[\hat{\delta}_{1,j}\mathbf{1}_S\right]$ ($j=1,...,K$) are given by expressions (5) and (7), respectively, in Section 2.5 in the main paper. Further, $E[\hat{\theta}_{1,1}\mathbf{1}_S]=E\left[\hat{\delta}_{1,1}\mathbf{1}_S\right]$ and for j ($j=2,...,K$), $E[\hat{\theta}_{1,j}\mathbf{1}_S]=E\left[\hat{\delta}_{1,j}\mathbf{1}_S\right]+E\left[\hat{\theta}_{1,1}\mathbf{1}_S\right]/\sqrt{V_{1,1}/V_{1,j}}$.

4.2 Selecting all treatments that have log hazard ratios below a futility value

For the rule of selecting all treatments that have log HRs below a futility threshold b, without loss of generality, if treatments 1 and 2 are selected,

$$\text{Prob}(S) = \int_{-\infty}^{b}\int_{-\infty}^{b}\int_{b}^{\infty}\cdots\int_{b}^{\infty} f(\hat{\theta}_{1,1},...,\hat{\theta}_{1,K})d\hat{\theta}_{1,K}\cdots d\hat{\theta}_{1,2}d\hat{\theta}_{1,1}.$$

The limits of integrations are constants and so it is easy to computate Prob(S) using standard statistical packages. Following Kan and Robotti [3], for j ($j = 1,...,K$),

$$E\left[\hat{\theta}_{1,j}\mathbf{1}_S\right] = \theta_j\text{Prob}(S) + \boldsymbol{e}_j\Sigma_{\hat{\theta}_1}\boldsymbol{c}^*,$$

where $\boldsymbol{e}_j$ and $\Sigma_{\hat{\theta}_1}$ are as defined in Section 2.5 in the main paper, and $\boldsymbol{c}^*$ is a vector similar to $\boldsymbol{c}$ in Section 3. The only difference for matrices and vectors $\Sigma_{i,j}$, $\Sigma_{-i,-j}$, $\Sigma_{-i,(j)}$, $\Sigma_{(i),-j}$ and $\tilde{\Sigma}_i$ is that they are extracted from $\Sigma_{\hat{\theta}_1}$. Then in place of δ and δ in the expression we replace by θ and θ, respectively. However, for all j ($j = 1,...,K$), we define $\theta_j^* = \left(\frac{b-\theta_j}{\Sigma_{j,j}}\right)$. The first and second elements in $\boldsymbol{c}^*$ are given by

$$-\frac{1}{\Sigma_{1,1}}\phi(\theta_1^*)\int_{-\infty}^{b}\int_{b}^{\infty}...\int_{b}^{\infty} f(\hat{\theta}_{1,(1)})d\hat{\theta}_{1,2}^*...d\hat{\theta}_{1,K}^*$$

and

$$-\frac{1}{\Sigma_{2,2}}\phi(\theta_2^*)\int_{-\infty}^{b}\int_{b}^{\infty}...\int_{b}^{\infty} f(\hat{\theta}_{1,(2)})d\hat{\theta}_{1,1}^*d\hat{\theta}_{1,3}^*...d\hat{\theta}_{1,K}^*,$$

respectively, and the jth ($j = 3,...,K$) entry in $\boldsymbol{c}^*$ is given by

$$\frac{1}{\Sigma_{j,j}}\phi(\theta_j^*)\int_{-\infty}^{b}\int_{-\infty}^{b}\int_{b}^{\infty}...\int_{b}^{\infty} f(\hat{\theta}_{1,(j)})d\hat{\theta}_{1,1}^*...d\hat{\theta}_{1,j-1}^*d\hat{\theta}_{1,j+1}^*...d\hat{\theta}_{1,K}^*.$$

Note that with this selection rule, for any number of selected treatments, the first and second elements in $\boldsymbol{c}^*$ that we have described above demonstrate the general form of elements for selected treatments while other entries have the form of the elements we have given for a dropped treatment.

4.3 Selecting all treatments that have pairwise p-values below a futility value

For the case of continuing with all treatments whose pairwise p-value are below a futility threshold a, the computations are similar to those of the rule in 4.2. In computing Prob(S), for $j \in S$ the upper limit in the integration $\Phi^{-1}(a)/\sqrt{V_{1,j}}$ while for $j \in S^c$ the lower limit in the integration is $\Phi^{-1}(a)/\sqrt{V_{1,j}}$. For the expected values, we define $\theta_j^* = \left(\frac{\Phi^{-1}(a)/\sqrt{V_{1,j}}-\theta_j}{\Sigma_{j,j}}\right)$ and limits of integration are changed as in the case of Prob(S).

5 UMVCUEs for specific selection rules

5.1 All treatments that have log hazard below a pre-speficied value b

In the main paper, this corresponds to the first selection rule in Table 1. Note that from the sufficient statistic $\hat{\theta}_1^* = \hat{\theta}_{1,1} + \frac{\sigma_{1,1}^2}{\sigma_{2,1}^2}\hat{\theta}_{2,1}$,

$$\hat{\theta}_{1,1} = \hat{\theta}_1^* - \frac{\sigma_{1,1}^2}{\sigma_{2,1}^2}\hat{\theta}_{2,1} \tag{1}$$

so that $L_1 = -\infty < \hat{\theta}_{1,1} < b = W_1$ implies that

$$B_L = \frac{\sigma_{2,1}^2}{-\sigma_{1,1}^2}(b - \hat{\theta}_1^*) < \hat{\theta}_{2,1} < \infty = B_U.$$

Re-expressing B_L we have

$$\begin{aligned} B_L &= \frac{\sigma_{2,1}^2}{-\sigma_{1,1}^2}\left(b - \frac{\sigma_{2,1}^2\hat{\theta}_{1,1} + \sigma_{1,1}^2\hat{\theta}_{2,1}}{\sigma_{2,1}^2}\right) = \frac{\sigma_{2,1}^2}{-\sigma_{1,1}^2}\left(b - \frac{\sigma_{1,1}^2 + \sigma_{2,1}^2}{\sigma_{2,1}^2}\frac{\sigma_{2,1}^2\hat{\theta}_{1,1} + \sigma_{1,1}^2\hat{\theta}_{2,1}}{\sigma_{1,1}^2 + \sigma_{2,1}^2}\right) \\ &= \frac{(\sigma_{1,1}^2 + \sigma_{2,1}^2)\hat{\theta}_1 - \sigma_{2,1}^2 b}{\sigma_{1,1}^2} \end{aligned}$$

so that

$$B_L - \hat{\theta}_1 = \frac{\sigma_{2,1}^2(\hat{\theta}_1 - b)}{\sigma_{1,1}^2}$$

and

$$\frac{B_L - \hat{\theta}_1}{\eta} = \frac{\sqrt{\sigma_{1,1}^2 + \sigma_{2,1}^2}}{\sigma_{2,1}^2} \times \frac{\sigma_{2,1}^2(\hat{\theta}_1 - b)}{\sigma_{1,1}^2} = \frac{\sqrt{\sigma_{1,1}^2 + \sigma_{2,1}^2}}{\sigma_{1,1}^2}\left(\hat{\theta}_1 - b\right).$$

Since $B_U = \infty$, $\phi\left(\frac{B_U - \hat{\theta}_1}{\eta}\right) = 0$ and $\Phi\left(\frac{B_U - \hat{\theta}_1}{\eta}\right) = 1$ so that expression (12) in the main paper give the UMVCUE for this selection rule.

5.2 All treatments that have pairwise p-value less than a pre-speficied value a

In the main paper, this corresponds to the second selection rule in Table 1. In this case, treatment 1 continues to stage 2 if $L_1 = -\infty < \hat{\theta}_{1,1} < \Phi^{-}(a)/\sqrt{V_{1,1}} = W_1$. Substituting b with $\Phi^{-}(a)/\sqrt{V_{1,1}}$ in Section 5.1, we observe that expression (12) in the main paper give the UMVCUE for this selection rule.

5.3 Treatment with the smallest pairwise p-value less than a pre-speficied value a

In the main paper, this corresponds to the fourth selection rule in Table 1. We assume treatment one is selected and for simplicity assume that $W_1 = \hat{\theta}_{1,2}\sqrt{V_{1,2}/V_{1,1}} = \hat{\theta}_{1,2}\sigma_{1,1}/\sigma_{1,2}$ so that W_1 corresponds to treatment two. Note that from the sufficient statistic $\hat{\theta}_2^* = \hat{\theta}_{1,2} + \frac{q_{12}}{\sigma_{2,1}^2}\hat{\theta}_{2,1}$,

$$\hat{\theta}_{1,2} = \hat{\theta}_2^* - \frac{q_{12}}{\sigma_{2,1}^2}\hat{\theta}_{2,1}$$

so that together with expression (1), $L_1 = -\infty < \hat{\theta}_{1,1} < \hat{\theta}_{1,2}\sigma_{1,1}/\sigma_{1,2} = W_1$ implies that

$$-\infty < \hat{\theta}_1^* - \frac{\sigma_{1,1}^2}{\sigma_{2,1}^2}\hat{\theta}_{2,1} < \frac{\sigma_{1,1}}{\sigma_{1,2}}\left(\hat{\theta}_2^* - \frac{q_{12}}{\sigma_{2,1}^2}\hat{\theta}_{2,1}\right)$$

and

$$-\infty < \left(\frac{q_{12}}{\sigma_{1,2}} - \sigma_{1,1}\right)\hat{\theta}_{2,1} < \left(\frac{\hat{\theta}_2^*}{\sigma_{1,2}} - \frac{\hat{\theta}_1^*}{\sigma_{1,1}}\right)\sigma_{2,1}^2.$$

If $q_{12} > \sigma_{1,1}\sigma_{1,2}$

$$B_L = -\infty < \hat{\theta}_{2,1} < \frac{\hat{\theta}_2^*/\sigma_{1,2} - \hat{\theta}_1^*/\sigma_{1,1}}{q_{12}/\sigma_{1,2} - \sigma_{1,1}} = B_U. \tag{2}$$

We simpltify B_U further. Noting that

$$\hat{\theta}_2^* = \frac{\sigma_{2,1}^2\hat{\theta}_{1,2} + q_{12}\hat{\theta}_{2,1}}{\sigma_{2,1}^2} \quad \text{and} \quad \hat{\theta}_1^* = \frac{\sigma_{1,1}^2 + \sigma_{2,1}^2}{\sigma_{2,1}^2}\hat{\theta}_1,$$

and susbstituting gives

$$B_U = \frac{\sigma_{1,1}(\sigma_{2,1}^2\hat{\theta}_{1,2} + q_{12}\hat{\theta}_{2,1}) - \sigma_{1,2}(\sigma_{1,1}^2 + \sigma_{2,1}^2)\hat{\theta}_1}{\sigma_{1,1}q_{12} - \sigma_{1,2}\sigma_{1,1}^2}.$$

Consequently,

$$\begin{aligned}
\frac{B_U - \hat{\theta}_1}{\eta} &= \frac{\sqrt{\sigma_{1,1}^2 + \sigma_{2,1}^2}}{\sigma_{2,1}^2}\left(\frac{\sigma_{1,1}(\sigma_{2,1}^2\hat{\theta}_{1,2} + q_{12}\hat{\theta}_{2,1}) - \sigma_{1,2}(\sigma_{1,1}^2 + \sigma_{2,1}^2)\hat{\theta}_1 - (\sigma_{1,1}q_{12} - \sigma_{1,2}\sigma_{1,1}^2)\hat{\theta}_1}{\sigma_{1,1}q_{12} - \sigma_{1,2}\sigma_{1,1}^2}\right) \\
&= \frac{\sqrt{\sigma_{1,1}^2 + \sigma_{2,1}^2}}{\sigma_{2,1}^2}\left(\frac{\sigma_{1,1}(\sigma_{2,1}^2\hat{\theta}_{1,2} + q_{12}\hat{\theta}_{2,1}) - (\sigma_{1,2}\sigma_{2,1}^2 + \sigma_{1,1}q_{12})\hat{\theta}_1}{\sigma_{1,1}q_{12} - \sigma_{1,2}\sigma_{1,1}^2}\right) \\
&= \frac{\sqrt{\sigma_{1,1}^2 + \sigma_{2,1}^2}}{\sigma_{2,1}^2}\left(\frac{(\sigma_{2,1}^2\sigma_{1,2} + \sigma_{1,1}q_{12})\hat{\theta}_1 - (\sigma_{2,1}^2\sigma_{1,2}(\sigma_{1,1}/\sigma_{1,2})\hat{\theta}_{1,2} + \sigma_{1,1}q_{12}\hat{\theta}_{2,1})}{\sigma_{1,2}\sigma_{1,1}^2 - \sigma_{1,1}q_{12}}\right) \\
&= \frac{(\sigma_{2,1}^2\sigma_{1,2} + \sigma_{1,1}q_{12})\hat{\theta}_1 - (\sigma_{2,1}^2\sigma_{1,2}W_1 + \sigma_{1,1}q_{12}\hat{\theta}_{2,1})}{\left(\sigma_{2,1}^2/\sqrt{\sigma_{1,1}^2 + \sigma_{2,1}^2}\right)(\sigma_{1,2}\sigma_{1,1}^2 - \sigma_{1,1}q_{12})}.
\end{aligned}$$

On the other hand since $B_L = -\infty$

$$\frac{B_L - \hat{\theta}_1}{\eta} = -\infty.$$

If $q_{12} < \sigma_{1,1}\sigma_{1,2}$

$$B_L = \frac{\hat{\theta}_2^*/\sigma_{1,2} - \hat{\theta}_1^*/\sigma_{1,1}}{q_{12}/\sigma_{1,2} - \sigma_{1,1}} < \hat{\theta}_{2,1} < \infty = B_U. \tag{3}$$

Substituting as above

$$\frac{B_L - \hat{\theta}_1}{\eta} = \frac{(\sigma_{2,1}^2\sigma_{1,2} + \sigma_{1,1}q_{12})\hat{\theta}_1 - (\sigma_{2,1}^2\sigma_{1,2}W_1 + \sigma_{1,1}q_{12}\hat{\theta}_{2,1})}{\left(\sigma_{2,1}^2/\sqrt{\sigma_{1,1}^2 + \sigma_{2,1}^2}\right)(\sigma_{1,2}\sigma_{1,1}^2 - \sigma_{1,1}q_{12})}$$

and

$$\frac{B_U - \hat{\theta}_1}{\eta} = \infty.$$

From these expressions for $\frac{B_L - \hat{\theta}_1}{\eta}$ and $\frac{B_U - \hat{\theta}_1}{\eta}$, expression (13) in the main paper and corresponding expressions for A_1 and A_2 (Row 2 in Table 2 in the main paper) give the UMVCUE when treatment with the smallest p-value is selected.

5.4 Treatment with the smallest log hazard ratio

In the main paper, this corresponds to the third selection rule in Table 1. To obtain expressions for for $\frac{B_L - \hat{\theta}_1}{\eta}$ and $\frac{B_U - \hat{\theta}_1}{\eta}$ for this rule, one can follow similar steps to that in Section 5.3. Alternatively, one can note if $\sigma_{1,1}^2 = ... = \sigma_{1,K}^2$, this selection rule is same as the selection rule in Section 5.3. So, in the derivation in Section 5.3, we susbstitute $\sigma_{1,2}$ with $\sigma_{1,1}$. Consequently, to decide whether to use inequality (2) or (3), q_{12} is compared to $\sigma_{1,1}^2$. Also,

$$\begin{aligned}\frac{(\sigma_{2,1}^2\sigma_{1,2} + \sigma_{1,1}q_{12})\hat{\theta}_1 - (\sigma_{2,1}^2\sigma_{1,2}W_1 + \sigma_{1,1}q_{12}\hat{\theta}_{2,1})}{\left(\sigma_{2,1}^2/\sqrt{\sigma_{1,1}^2 + \sigma_{2,1}^2}\right)(\sigma_{1,2}\sigma_{1,1}^2 - \sigma_{1,1}q_{12})} &= \frac{\sigma_{1,1}[(\sigma_{2,1}^2 + q_{12})\hat{\theta}_1 - (\sigma_{2,1}^2W_1 + q_{12}\hat{\theta}_{2,1})]}{\sigma_{1,1}\left(\sigma_{2,1}^2/\sqrt{\sigma_{1,1}^2 + \sigma_{2,1}^2}\right)(\sigma_{1,1}^2 - q_{12})} \\ &= \frac{(\sigma_{2,1}^2 + q_{12})\hat{\theta}_1 - (\sigma_{2,1}^2W_1 + q_{12}\hat{\theta}_{2,1})}{\left(\sigma_{2,1}^2/\sqrt{\sigma_{1,1}^2 + \sigma_{2,1}^2}\right)(\sigma_{1,1}^2 - q_{12})}.\end{aligned}$$

Hence if $q_{12} > \sigma_{1,1}^2$,

$$\frac{B_U - \hat{\theta}_1}{\eta} = \frac{(\sigma_{2,1}^2 + q_{12})\hat{\theta}_1 - (\sigma_{2,1}^2W_1 + q_{12}\hat{\theta}_{2,1})}{\left(\sigma_{2,1}^2/\sqrt{\sigma_{1,1}^2 + \sigma_{2,1}^2}\right)(\sigma_{1,1}^2 - q_{12})}$$

and

$$\frac{B_L - \hat{\theta}_1}{\eta} = -\infty,$$

Hence if $q_{12} < \sigma_{1,1}^2$,

$$\frac{B_L - \hat{\theta}_1}{\eta} = \frac{(\sigma_{2,1}^2 + q_{12})\hat{\theta}_1 - (\sigma_{2,1}^2 W_1 + q_{12}\hat{\theta}_{2,1})}{\left(\sigma_{2,1}^2 / \sqrt{\sigma_{1,1}^2 + \sigma_{2,1}^2}\right)(\sigma_{1,1}^2 - q_{12})}$$

and

$$\frac{B_U - \hat{\theta}_1}{\eta} = \infty,$$

so that the UMVCUE for this selection is given by expression (13) in the main paper and corresponding expressions for A_1 and A_2 (Row 1 in Table 2 in the main paper).

5.5 Other selection rules in Table 1 in the main paper

The UMVCUEs for the last two selection rules in Table 1 follows the previous UMVCUEs. Taking the case of continuing with treatment with the smallest log hazard ratio $\leq b$, if $W_j = b$, the UMVCUE is given by expression (12) in the main paper, while if $W_j \neq b$ the UMVCUE is given by expression (13) in the main paper.

6 Iteration procedure for estimating ν^2 in the expression for the shrinkage estimator

The iteration procedure has been given by Brückner *et al* [4] and replicated by Kimani *et al* [2]. The procedure is faster computationally if the variance-covariance matrix is diagonal. From Section 3.2 in the main paper, we note that the posterior marginal variance-covariance matrix for θ is $\nu^2 \boldsymbol{I}_K + \Sigma_{\hat{\theta}_1}$. We seek a linear transformation U such that the posterior mean for $U\theta$ is $\nu^2 \boldsymbol{I}_K + D$, where D is a diagonal matrix. This is achieved by using the single value decomposition. Since $\Sigma_{\hat{\theta}_1}$ is symmetric, $D = U\Sigma_{\hat{\theta}_1}U'$ where D is the diagonal matrix of eigenvalues for $\Sigma_{\hat{\theta}_1}$ and U is an orthogonal matrix. Following Brückner *et al*, the iteration procedure is then to specify an initial guess $\hat{\nu}^2$ for ν^2 and repeat the following procedure until convergence

- Define weights $w_i = (\nu^2 + D_{ii}^2)^{-1}$ $(i = 1, ..., K)$.

- Update the estimated value for ν^2 as $\hat{\nu}^2 = \frac{\sum_{i=1}^K w_i[(\hat{\theta}_{1,i} - \hat{\theta}_{1,all}) - D_{ii}^2]}{\sum_{i=1}^K w_i}$.

Denoting the solution of the iteration procedure by $\tilde{\nu}^2$, the value of ν^2 used to compute the shrinkage estimator is $\tilde{\nu}_+^2 = \max\{0, \tilde{\nu}^2\}$.

7 Worked example

7.1 Template for the dataset

Table S3 shows key variables in the dataset we analysed. Each row corresponds to a unique patient. The variables are "EnterTime" (Study entry time in weeks with numbering starting from first recruited patient), "Stage" (1 for stage 1 patients and 0 for stage 2 patients), "Arm" (0 for control, 1 for

Treatment 1 which corresponds to mood stabiliser plus 24 weeks of atypical antipsychotic and 2 for Treatment 2 which corresponds to mood stabiliser plus 52 weeks of atypical antipsychotic), "T.date" (at the end of the study, survival time in weeks), "Relapse" (at end of study, 1 if relapse and 0 if no relapse), "T.date1" (at the time of interim analysis, survival time in weeks for stage 1 patients) and "Relapse1" (at interim analysis time, 1 if relapse and 0 if no relapse).

Table S3: Template dataset

EnterTime	Stage	Arm	T.date	Relapse	T.date1	Relapse1
1	1	1	27.739191	1	27.739191	1
2	1	0	27.575033	1	27.575033	1
3	1	2	52.347972	0	52.347972	0
4	1	1	1.556884	1	1.556884	1
5	1	0	21.770252	0	21.770252	0
6	1	2	16.685259	1	16.685259	1
7	1	1	15.980670	1	15.980670	1
8	1	2	52.347972	0	52.347972	0
9	1	0	15.025232	0	15.025232	0
10	1	2	5.928287	1	5.928287	1
.	.	.	.	.	.	.
.	.	.	.	.	.	.
.	.	.	.	.	.	.
150	0	1	40.189391	1	·	·
151	0	2	51.758591	0	·	·
152	0	1	48.996754	1	·	·
153	0	0	11.705179	1	·	·
154	0	1	54.461218	0	·	·
155	0	0	29.035857	1	·	·
156	0	2	6.685259	0	·	·
157	0	1	57.171476	0	·	·
158	0	2	1.960159	1	·	·
159	0	1	15.980670	1	·	·

7.2 Computing single iteration estimates: expression for expected and parameter values for the density

The single iteration bias adjusted estimator assumes the naive estimate are the true values so that joint density of stage 1 log HRs $f(\hat{\theta}_{1,1}, \hat{\theta}_{1,2})$ is

$$\begin{pmatrix} \hat{\theta}_{1,1} \\ \hat{\theta}_{1,2} \end{pmatrix} \sim MVN\left(\begin{pmatrix} \theta_1 \\ \theta_2 \end{pmatrix}, \begin{pmatrix} \sigma_{1,1}^2 & \nu_{1,2}\sigma_{1,1}^2\sigma_{1,2}^2 \\ \nu_{1,2}\sigma_{1,1}^2\sigma_{1,2}^2 & \sigma_{1,2}^2 \end{pmatrix} \right) = MVN\left(\begin{pmatrix} -0.6528 \\ -0.5796 \end{pmatrix}, \begin{pmatrix} 0.1239 & 0.0522 \\ 0.0522 & 0.1146 \end{pmatrix} \right). \quad (4)$$

Following Section 4.3 in this supplementary document, the expression for probability of continuing with both Treatments 1 and 2 is

$$\text{Prob}(S) = \int_{-\infty}^{W_1} \int_{-\infty}^{W_2} f(\hat{\theta}_{1,1}, \hat{\theta}_{1,2}) d\hat{\theta}_{1,2} d\hat{\theta}_{1,1}.$$

Let $\boldsymbol{e}_1 = (1,0)'$, $\boldsymbol{e}_2 = (0,1)'$ and $\Sigma_{\hat{\theta}_1}$ the covariance matrix in expression (4). Following Section 4.3 in this supplementary document, the stage 1 expected value for log HR for Treatment j ($j = 1,2$)

$$E\left[\hat{\theta}_{1,j}\mathbf{1}_S\right] = \theta_j \text{Prob}(S) + \boldsymbol{e}_j \Sigma_{\hat{\theta}_1} \boldsymbol{c}^*,$$

where $\boldsymbol{c}^*$ is a two element vector whose values are obtained as follows. The only difference for matrices and vectors $\Sigma_{i,j}$, $\Sigma_{-i,-j}$, $\Sigma_{-i,(j)}$, $\Sigma_{(i),-j}$ and $\tilde{\Sigma}_i$ is that they are extracted from $\Sigma_{\hat{\theta}_1}$. Then in place of δ and δ in the expression we replace by θ and θ, respectively. For j ($j = 1,2$), we define $\theta_j^* = \left(\frac{W_j - \theta_j}{\Sigma_{j,j}}\right)$. The first and second elements in $\boldsymbol{c}^*$ are given by

$$-\frac{1}{\sigma_{1,1}}\phi\left(\frac{W_1-\theta_1}{\sigma_{1,1}^2}\right)\int_{-\infty}^{W_2} f(\hat{\theta}_{1,(1)}) d\hat{\theta}_{1,2}^* \quad \text{and} \quad -\frac{1}{\sigma_{1,2}}\phi\left(\frac{W_2-\theta_2}{\sigma_{1,2}^2}\right)\int_{-\infty}^{W_1} f(\hat{\theta}_{1,(2)}) d\hat{\theta}_{1,1}^*, \tag{5}$$

respectively, where $f(\hat{\theta}_{1,(1)})$ is the density of a normal distribution with mean $\theta_2 + q_{12}\left(\frac{W_1-\theta_1}{\sigma_{1,1}^2}\right)$ and variance $\sigma_{1,2}^2 - \frac{1}{\sigma_{1,1}^2}q_{12}^2$ and $f(\hat{\theta}_{1,(2)})$ is the density of a normal distribution with mean $\theta_1 + q_{12}\left(\frac{W_2-\theta_2}{\sigma_{1,2}^2}\right)$ and variance $\sigma_{1,1}^2 - \frac{1}{\sigma_{1,2}^2}q_{12}^2$.

7.3 Computing multiple iterations estimates

The function for computing multiple iterations estimates involves adding iteration step to the single iteration function provided in the repository "https://github.com/KimaniPK/Treatment-Selection-with-TTE-data". This includes updating probability of continuing with the two treatments. In iteration 1, naive estimates are used. This corresponds to the first object in the multiple iterations function. Bias is a function t_j as defined in expression (3) in the main paper and it is the second object (vector $(t_1,t_2)'$) in the multiple iterations function. As described in Section 2.5 in the main paper, bias is computed based on the multivariate joint density of stage 1 estimates. This corresponds to the third object to be input in the function. The fourth input for the function is the futility threshold value because bias is a function of futility rule.

8 Characteristics of simulated trials when an experimental treatment is selected

The median number of deaths from patients recruited in stage 1 and the number of simulated trials where there is no convergence while computing $\hat{\theta}_{j,MI}$ estimate are given in Table S4. The observed total stage 1 number of deaths from the control and selected experimental treatment is different from that expected with no treatment selection and stopping for futility (expected numbers are 159 in control and 139 in experimental treatments for scenario 2) suggesting possibility of bias. A high number of events is observed from stage 1 patients without events at the interim analysis which reflects substantial gain in reducing variability of estimates. In scenario 3, when a substantially inferior treatment is selected, while computing multiple iterations estimate $\hat{\theta}_{j,MI}$, convergence can fail almost 14 times in 1000 trials. For other scenarios, non-convergence is less than 6 in 1000 trials. The rate of non-convergence seems to be higher when true log HRs for treatments are close, the selected treatment is not the truly most effective and futility value is further from the true log HR.

Table S4: Characteristics of simulated trials when an experimental treatment is selected.

	Mean hazard ratios					Stage 1 deaths		Additional deaths		Non-convergent
Treatment	$\hat{\theta}_j$	$\hat{\theta}_{j,UMV}$	$\hat{\theta}_{j,MI}$	$\hat{\theta}_{j,SI}$	$\hat{\theta}_{j,SH}$	Control	Treatment j	Control	Treatment j	cases (%) for $\hat{\theta}_{j,MI}$
Scenario 1: True hazard ratios are $\exp(\theta_1) = \exp(\theta_2) = \exp(\theta_3) = \exp(\theta_4) = 1$										
1	0.95	1.00	1.02	0.99	0.97	145	135	28	30	16 (0.080)
2	0.95	1.00	1.02	0.99	0.97	145	135	28	30	39 (0.193)
3	0.95	1.00	1.02	0.99	0.97	145	135	28	30	24 (0.122)
4	0.95	1.00	1.02	0.99	0.97	145	135	28	30	39 (0.196)
Scenario 2: True hazard ratios are $\exp(\theta_1) = \exp(\theta_2) = \exp(\theta_3) = \exp(\theta_4) = 0.8$										
1	0.77	0.80	0.82	0.80	0.79	160	132	29	29	26 (0.106)
2	0.77	0.80	0.82	0.80	0.79	160	132	29	29	25 (0.100)
3	0.77	0.80	0.82	0.79	0.80	160	131	29	29	28 (0.111)
4	0.77	0.80	0.82	0.80	0.79	160	132	29	29	19 (0.076)
Scenario 3: True hazard ratios are $\exp(\theta_1) = 1$, $\exp(\theta_2) = 0.87$, $\exp(\theta_3) = 0.74$ and $\exp(\theta_4) = 0.6$										
2	0.79	0.86	0.87	0.82	0.79	160	128	29	32	0 (0.000)
3	0.70	0.74	0.75	0.72	0.72	161	121	29	29	4 (0.080)
4	0.60	0.60	0.61	0.60	0.61	161	114	29	25	0 (0.000)
Scenario 4: True hazard ratios are $\exp(\theta_1) = \exp(\theta_2) = \exp(\theta_3) = 0.4$ and $\exp(\theta_4) = 0.35$										
1	0.38	0.40	0.41	0.39	0.39	232	113	32	24	9 (0.086)
2	0.38	0.40	0.41	0.39	0.39	231	113	32	24	9 (0.087)
3	0.38	0.40	0.41	0.39	0.38	232	113	32	24	8 (0.077)
4	0.34	0.35	0.36	0.35	0.35	232	109	32	21	1 (0.001)

† j corresponds to the experimental treatment in Column 1. Each row corresponds to simulated trials where the experimental treatment in Column 1 is selected.

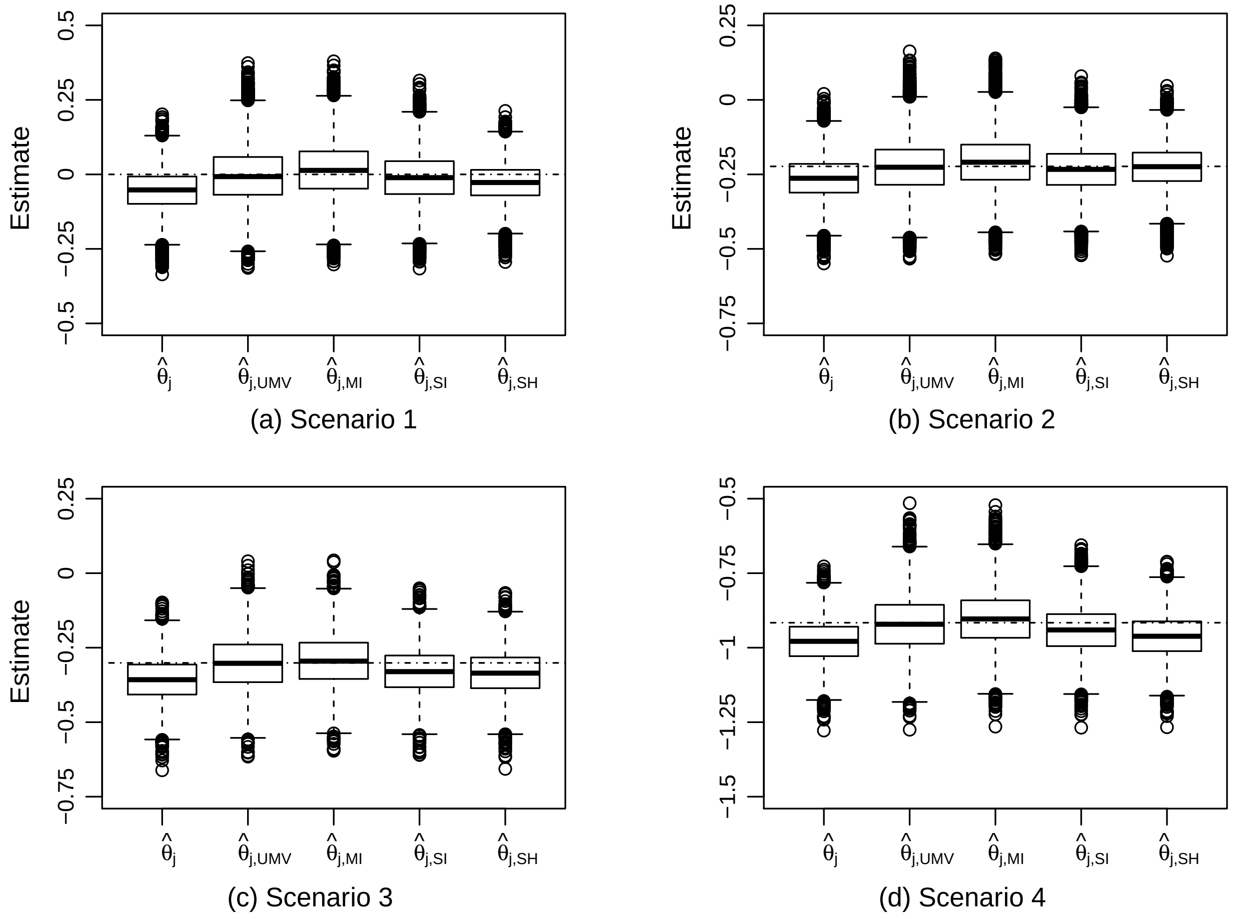

Figure S1: Boxplots for treatment three log hazard ratio estimates for scenarios 1 to 4. The true log hazard ratios are represented by the dashed and dotted lines.

.

SIMULATIONS WHEN THERE ARE TWO TREATMENTS IN STAGE 1

9 Simulation results for the case of two treatments in stage 1

We consider four scenarios. Treatment effects for treament two in scenarios 1 to 4 are equal to those of treatment four in the main results (Section 6 in the main paper) in scenarios 1 to 4, respectively. Treatment effects for treatment one in scenarios 1 to 4 are equal to those of treatment three in the main results in scenarios 1 to 4, respectively. Therefore properties of estimates for treatments two and one for the case of two treatments (this section) can be compared to properties of estimates for treatments four and three for the case of four treatments (Section 6 in the main paper), respectively. Sample size of the simulated studies are similar to those for the case of four treatments in stage 1, that is, interim analysis after 437 events (159 in control and 139 in each experimental treatment), 316 events in stage 2 and following stage 1 patients for one year after the interim analysis.

The results for the case of two treatments in stage 1 are summarised in Tables S5 and S6 and Figures S2 and S3. Compared to the case of four treatments in stage 1, the biases are smaller and there are few cases of non-convergence for multiple iterations bias subtracted estimator. This is expected from previous research [5–7]. Findings for comparing different estimators are similar to those for the case of four treatments in stage 1.

Table S5: Simulated biases and root mean squared errors for the various estimators for the log hazard ratios for the case of two treatments in stage 1

Treatment	Selection probability	Simulated bias					Root mean squared error				
		$\hat{\theta}_j$	$\hat{\theta}_{j,UMV}$	$\hat{\theta}_{j,MI}$	$\hat{\theta}_{j,SI}$	$\hat{\theta}_{j,SH}$	$\hat{\theta}_j$	$\hat{\theta}_{j,UMV}$	$\hat{\theta}_{j,MI}$	$\hat{\theta}_{j,SI}$	$\hat{\theta}_{j,SH}$
Scenario 1: True log hazard ratios are $\theta_1 = \theta_2 = 0$											
1	0.3358	-0.0451	**-0.0037**	0.0094	-0.0092	-0.0364	0.0806	0.0909	0.0905	0.0815	**0.0731**
2	0.3325	-0.0446	**-0.0032**	0.0099	-0.0087	-0.0363	0.0797	0.0900	0.0896	0.0806	**0.0725**
Scenario 2: True log hazard ratios are $\theta_1 = \theta_2 = -0.2231$											
1	0.4996	-0.0217	**-0.0004**	0.0095	-0.0055	-0.0102	0.0762	0.0836	0.0850	0.0778	**0.0723**
2	0.4952	-0.0214	**0.0000**	0.0099	-0.0051	-0.0101	0.0759	0.0836	0.0850	0.0776	**0.0722**
Scenario 3: True log hazard ratios are $\theta_1 = -0.3011$ and $\theta_2 = -0.5108$											
1	0.0449	-0.0548	**0.0016**	0.0084	-0.0286	-0.0520	0.0907	0.0902	0.0888	**0.0807**	0.0880
2	0.9551	-0.0031	**-0.0003**	0.0045	0.0017	0.0141	**0.0770**	0.0792	0.0810	0.0788	0.0785
Scenario 4: True log hazard ratios are $\theta_1 = -0.9163$ and $\theta_2 = -1.0498$											
1	0.1636	-0.0489	**-0.0012**	0.0077	-0.0241	-0.0387	0.0906	0.0940	0.0923	**0.0832**	0.0840
2	0.8364	-0.0107	-0.0009	0.0074	**-0.0005**	0.0063	0.0811	0.0873	0.0892	0.0836	**0.0798**

Table S6: Characteristics of simulated trials when an experimental treatment is selected for the case of two treatments in stage 1

Treatment	Mean hazard ratios					Stage 1 deaths		Additional deaths		Non-convergent
	$\hat{\theta}_j$	$\hat{\theta}_{j,UMV}$	$\hat{\theta}_{j,MI}$	$\hat{\theta}_{j,SI}$	$\hat{\theta}_{j,SH}$	Control	Treatment j	Control	Treatment j	cases (%) for $\hat{\theta}_{j,MI}$
Scenario 1: True hazard ratios are $\exp(\theta_1) = \exp(\theta_2) = 1$										
1	0.96	1.00	1.01	0.99	0.97	149	139	39	42	2 (0.006)
2	0.96	1.00	1.01	0.99	0.97	149	139	39	42	3 (0.009)
Scenario 2: True hazard ratios are $\exp(\theta_1) = \exp(\theta_2) = 0.8$										
1	0.78	0.80	0.81	0.8	0.79	161	134	41	39	0 (0)
2	0.79	0.80	0.81	0.8	0.79	161	133	41	39	0 (0)
Scenario 3: True hazard ratios are $\exp(\theta_1) = 0.74$ and $\exp(\theta_2) = 0.6$										
1	0.70	0.74	0.75	0.72	0.70	175	129	42	40	0 (0)
2	0.60	0.60	0.60	0.60	0.61	174	121	43	35	0 (0)
Scenario 4: True hazard ratios are $\exp(\theta_1) = 0.40$ and $\exp(\theta_2) = 0.35$										
1	0.38	0.40	0.40	0.39	0.39	221	105	29	22	0 (0)
2	0.35	0.35	0.35	0.35	0.35	220	101	29	19	0 (0)

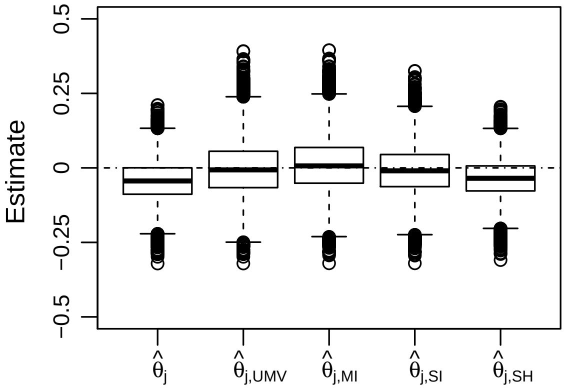

(a) Scenario 1

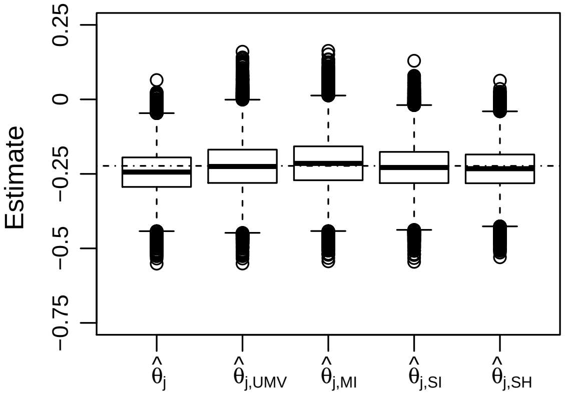

(b) Scenario 2

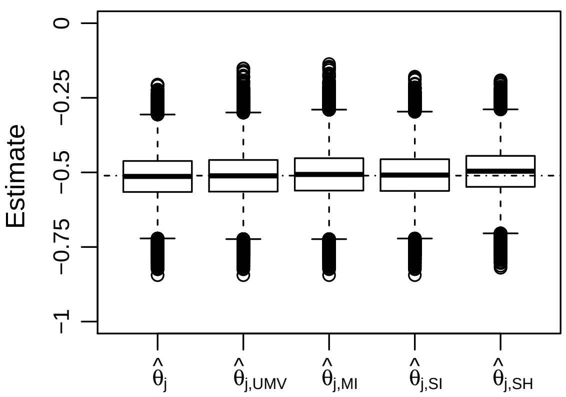

(c) Scenario 3

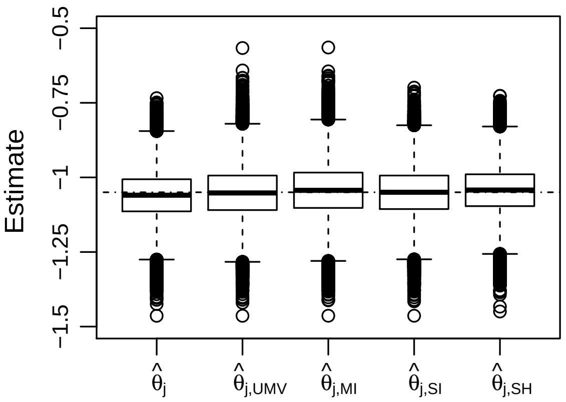

(d) Scenario 4

Figure S2: Boxplots for treatment two log hazard ratio estimates for scenarios 1 to 4 for the case of two experimental treatments in stage 1. The true log hazard ratios are represented by the dashed and dotted lines.

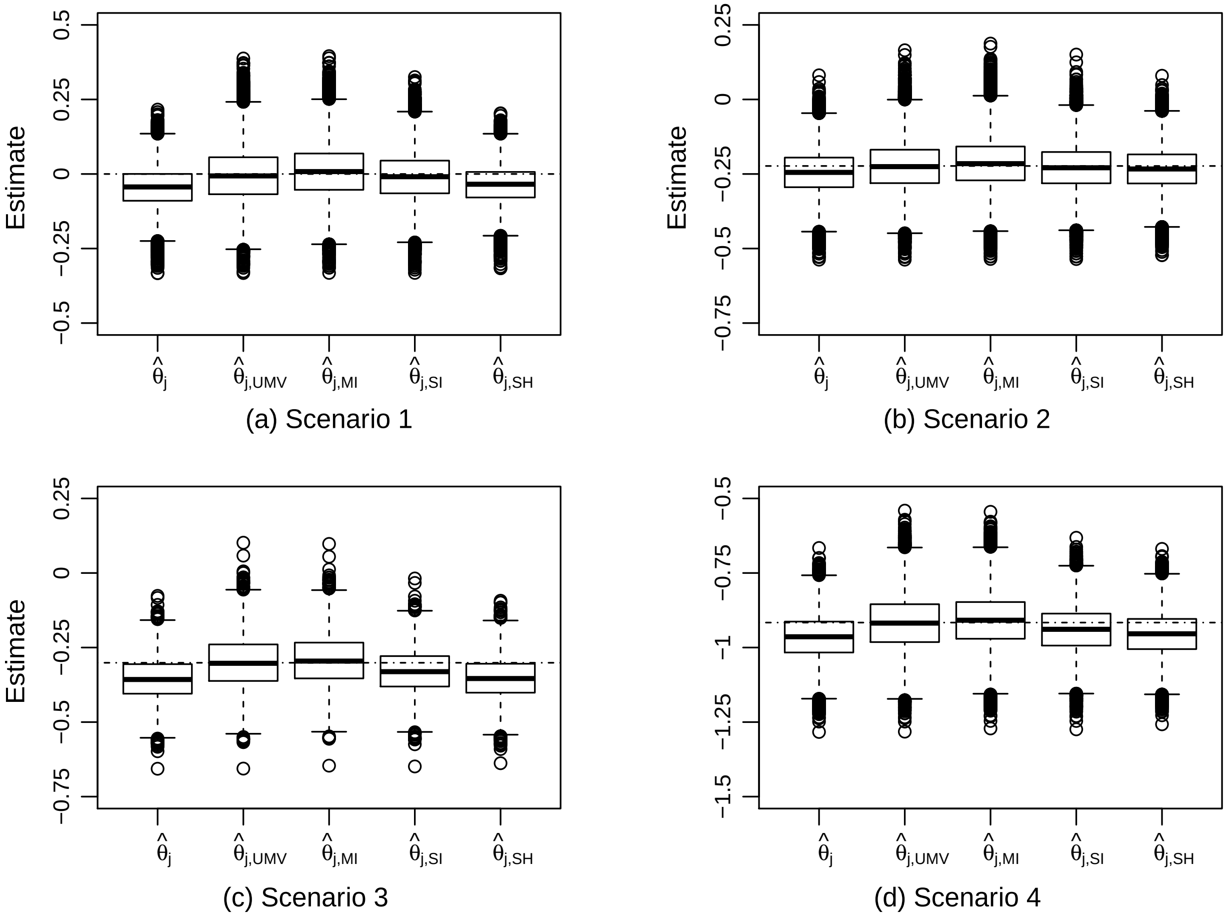

Figure S3: Boxplots for treatment one log hazard ratio estimates for scenarios 1 to 4 for the case of two experimental treatments in stage 1. The true log hazard ratios are represented by the dashed and dotted lines.

SIMULATIONS WITH A SMALLER SAMPLE SIZE
AND
FOUR TREATMENTS IN STAGE ONE

10 Simulation results for the case of smaller sample size

In this section, we present the results for the case of four treatments in stage 1 and when the trial is designed such that the hazard ratio 0.6. Consequently few deaths and hence sample size is required.

Sample sizes in simulations are such that, if it was a single-stage trial and the true hazard ratio (HR) is 0.6, the combined stage 1 and stage 2 number of deaths from the control and the selected experimental treatment correspond to power of 80%. For 80% power, 122 deaths (rounded up to an even number) are required. We fixed deaths from patients recruited in stage 2, that is stage 2 patients, to be 61.

Recalling that we take the shape parameter $\gamma = 0.5$ and taking time t in the hazard function to be in days, we set the scale parameter for the control treatment such that the median is 365 days, that is, $\lambda_0 = \ln(2)/365$. We determined the number of deaths in stage 1 assuming the four HRs corresponding to the four experimental treatments are all equal to 0.6, that is, $\lambda_i = 0.6\lambda_0$ $(i = 1, \ldots, 4)$. The scale parameter for the experimental treatment can be computed from the HR and the scale parameter for the control. Assuming that on average 1 person is recruited every four days in stage 1, we set the interim analysis to be performed after 146 deaths which, after rounding off, corresponds to the expected number of events comparing each experimental treatment to the control to be 65 (38 in control and 27 in the experimental treatments) when hazard ratios are all equal to 0.6.

Assuming a uniform recruitment rate of 1 patient every four days in stage 2, for the assumed hazard functions when HR is 0.6, we expect 61 events from stage 2 patients to be observed after approximately 2 years. Therefore we fixed to follow the stage 1 patients from the control and selected experimental treatment without events at the interim analysis to be followed for 1 year post interim analysis.

Simulations are based on the same four scenarios. For easier comparisons, the captions of the tables and the figures in this section indicate corresponding results based on the bigger sample size. Compared to the case with larger sample sizes, there are more cases for non-convergence for multiple iterations bias subtracted estimator (Table S8). Also, the biases and RMSEs are larger (Table S7 and Figures S4 and S5). However, comparing the various estimators leads to the same conclusion as in the case of bigger sample size.

Table S7: Simulated biases and root mean squared errors for the various estimators for the log hazard ratios for the case of smaller sample size. **(Compare with results in Table 4 in the main paper.)**

	Selection	Simulated bias					Root mean squared error				
Treatment	probability	$\hat{\theta}_j$	$\hat{\theta}_{j,UMV}$	$\hat{\theta}_{j,MI}$	$\hat{\theta}_{j,SI}$	$\hat{\theta}_{j,SH}$	$\hat{\theta}_j$	$\hat{\theta}_{j,UMV}$	$\hat{\theta}_{j,MI}$	$\hat{\theta}_{j,SI}$	$\hat{\theta}_{j,SH}$
Scenario 1: True log hazard ratios are $\theta_1 = \theta_2 = \theta_3 = \theta_4 = 0$											
1	0.2016	-0.1072	**-0.0086**	0.0229	-0.0199	-0.0726	0.1816	0.1897	0.1883	0.1735	**0.1533**
2	0.1974	-0.1027	**-0.0041**	0.0274	-0.0151	-0.0785	0.1778	0.1878	0.1874	0.1717	**0.1558**
3	0.1992	-0.1024	**-0.0026**	0.0285	-0.0143	-0.0665	0.1788	0.1890	0.1887	0.1731	**0.1479**
4	0.2000	-0.1044	**-0.0062**	0.0247	-0.0172	-0.0794	0.1796	0.1890	0.1878	0.1729	**0.1572**
Scenario 2: True log hazard ratios are $\theta_1 = \theta_2 = \theta_3 = \theta_4 = -0.2231$											
1	0.2422	-0.0838	**-0.0016**	0.0281	-0.0143	-0.0421	0.1735	0.1840	0.1853	0.1697	**0.1502**
2	0.2428	-0.0841	**-0.0024**	0.0274	-0.0149	-0.0517	0.1734	0.1827	0.1844	0.1691	**0.1545**
3	0.2414	-0.0831	**-0.0008**	0.0290	-0.0135	-0.0263	0.1732	0.1843	0.1857	0.1696	**0.1458**
4	0.2426	-0.0854	**-0.0038**	0.0258	-0.0162	-0.0528	0.1755	0.1848	0.1860	0.1711	**0.1567**
Scenario 3: True log hazard ratios are $\theta_1 = 0$, $\theta_2 = -0.1393$, $\theta_3 = -0.3011$ and $\theta_4 = -0.5108$											
1	0.0123	-0.1611	-0.0107	**0.0053**	-0.0602	-0.1546	0.2142	0.1826	0.1741	**0.1719**	0.2037
2	0.0544	-0.1231	**-0.0069**	0.0153	-0.0414	-0.1079	0.1956	0.1845	0.1817	**0.1732**	0.1837
3	0.2072	-0.0826	**-0.0016**	0.0216	-0.0216	-0.0228	0.1753	0.1832	0.1838	0.1700	**0.1602**
4	0.7138	-0.0328	**-0.0015**	0.0221	0.0031	0.0234	0.1661	0.1796	0.1846	0.1742	**0.1662**
Scenario 4: True log hazard ratios are $\theta_1 = \theta_2 = \theta_3 = -0.9163$ and $\theta_4 = -1.0498$											
1	0.1875	-0.1089	**-0.0103**	0.0201	-0.0349	-0.0644	0.1909	0.1902	0.1881	0.1727	**0.1613**
2	0.1897	-0.1090	**-0.0111**	0.0190	-0.0355	-0.0740	0.1907	0.1895	0.1873	0.1725	**0.1677**
3	0.1869	-0.1079	**-0.0089**	0.0211	-0.0340	-0.0629	0.1906	0.1900	0.1885	**0.1728**	0.1744
4	0.4359	-0.0733	**-0.0067**	0.0242	-0.0148	-0.0264	0.1788	0.1912	0.1923	0.1757	**0.1619**

Table S8: Additional information of the simulation study for sample size. **(Compare with results in Table S4 in this document.)**

Treatment	Mean hazard ratios					Stage 1 deaths		Additional deaths		Non-convergent
	$\hat{\theta}_j$	$\hat{\theta}_{j,UMV}$	$\hat{\theta}_{j,MI}$	$\hat{\theta}_{j,SI}$	$\hat{\theta}_{j,SH}$	Control	Treatment j	Control	Treatment j	cases (%) for $\hat{\theta}_{j,MI}$
Scenario 1: True hazard ratios are $\exp(\theta_1) = \exp(\theta_2) = \exp(\theta_3) = \exp(\theta_4) = 1$										
1	0.91	1.01	1.04	1.00	0.94	30	25	15	17	67 (0.332)
2	0.91	1.01	1.05	1.00	0.93	30	25	15	17	60 (0.304)
3	0.91	1.02	1.05	1.00	0.94	30	25	15	17	73 (0.366)
4	0.91	1.01	1.04	1.00	0.93	30	25	15	17	71 (0.355)
Scenario 2: True hazard ratios are $\exp(\theta_1) = \exp(\theta_2) = \exp(\theta_3) = \exp(\theta_4) = 0.8$										
1	0.74	0.81	0.84	0.80	0.78	34	24	16	16	49 (0.202)
2	0.74	0.81	0.84	0.80	0.77	34	24	16	16	39 (0.161)
3	0.74	0.81	0.84	0.80	0.79	34	24	16	16	40 (0.166)
4	0.74	0.81	0.84	0.80	0.77	34	24	17	16	48 (0.198)
Scenario 3: True hazard ratios are $\exp(\theta_1) = 1$, $\exp(\theta_2) = 0.87$, $\exp(\theta_3) = 0.74$ and $\exp(\theta_4) = 0.60$										
1	0.86	1.01	1.02	0.95	0.86	34	25	16	19	34 (2.764)
2	0.78	0.88	0.90	0.85	0.79	34	24	16	17	37 (0.680)
3	0.69	0.75	0.77	0.73	0.73	34	22	16	15	12 (0.058)
4	0.59	0.61	0.62	0.61	0.62	34	21	17	13	4 (0.006)
Scenario 4: True hazard ratios are $\exp(\theta_1) = \exp(\theta_2) = \exp(\theta_3) = 0.40$ and $\exp(\theta_4) = 0.35$										
1	0.36	0.40	0.42	0.39	0.38	51	20	20	12	12 (0.064)
2	0.36	0.40	0.41	0.39	0.38	51	20	20	12	15 (0.079)
3	0.36	0.40	0.42	0.39	0.38	51	20	20	12	10 (0.054)
4	0.33	0.35	0.37	0.35	0.35	51	19	20	11	8 (0.018)

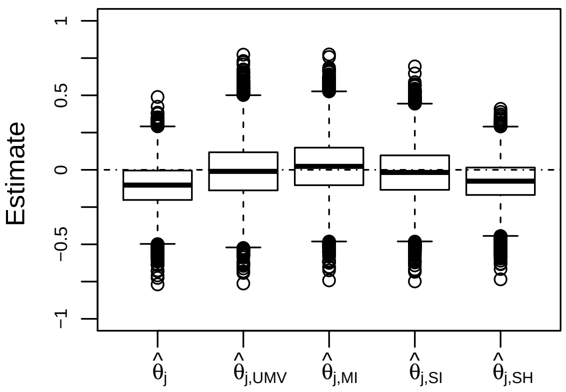

(a) Scenario 1

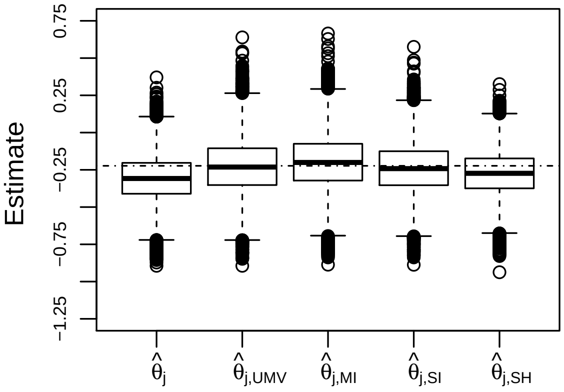

(b) Scenario 2

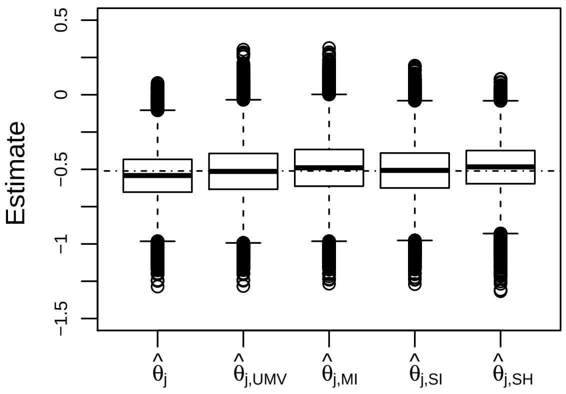

(c) Scenario 3

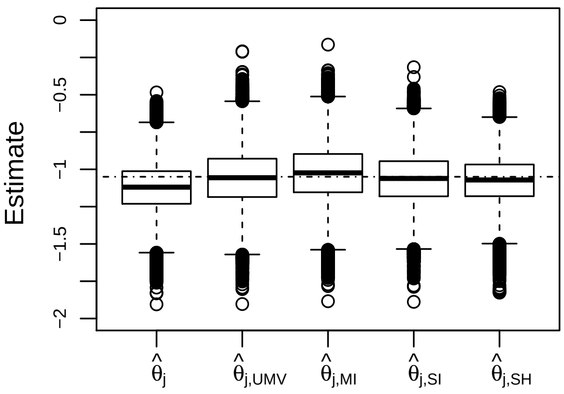

(d) Scenario 4

Figure S4: Boxplots for treatment one log hazard ratio estimates for scenarios 1 to 4 for the case of two experimental treatments in stage 1. The true log hazard ratios are represented by the dashed and dotted lines. **(Compare with Figure 2 in the main paper. Note different y-axes scales.)**

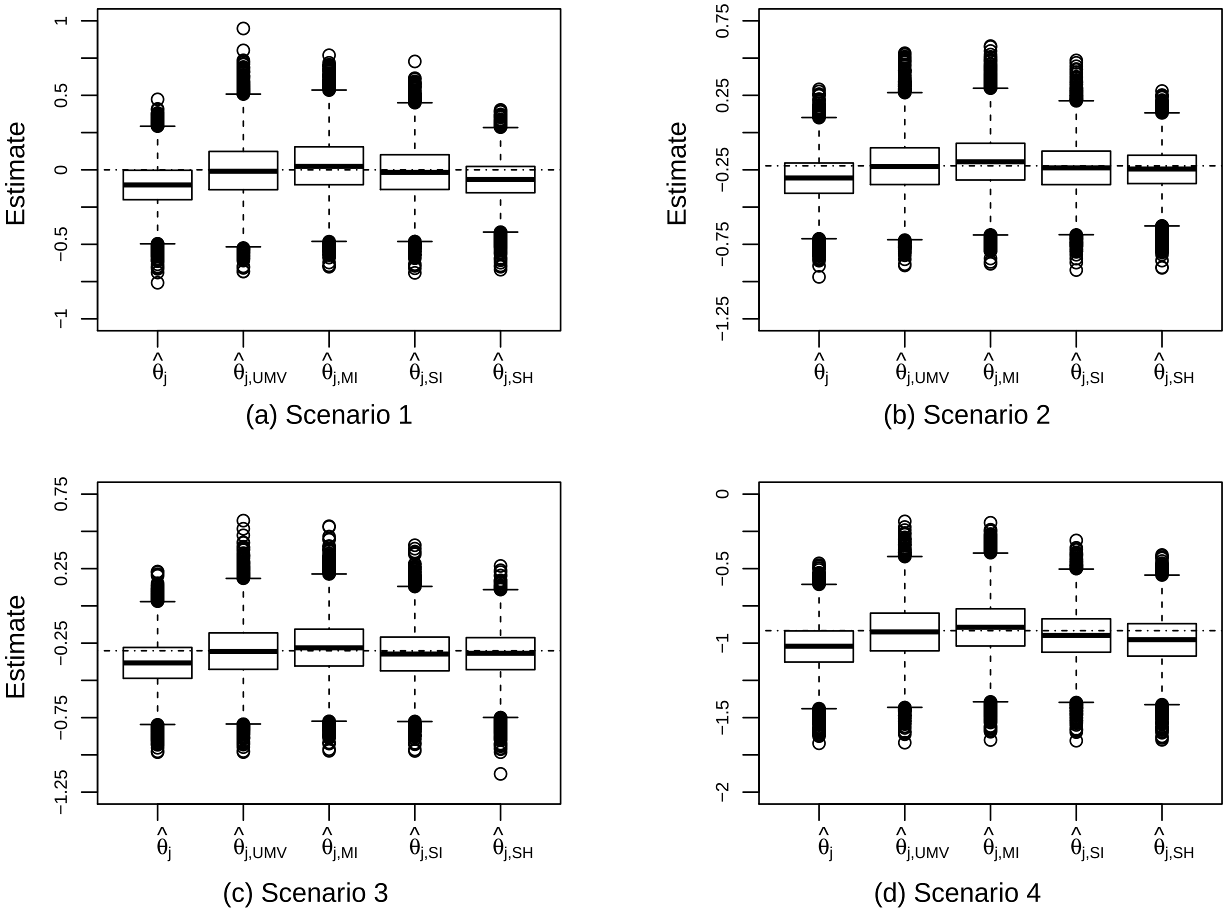

Figure S5: Boxplots for treatment one log hazard ratio estimates for scenarios 1 to 4 for the case of two experimental treatments in stage 1. The true log hazard ratios are represented by the dashed and dotted lines. **(Compare with Figure S1 in this document. Note different y-axes scales.)**

.

COMPUTATIONS BASED ON LOGRANK SCORE STATISTIC

11 Computations based on logrank score statistic

In this section (rest of this document), we present computation results based on logrank score statistic. For easier comparisons, the captions of the tables and the figures in this section indicate corresponding results based on logrank score statistic.

11.1 Worked example

The constructed phase II/III clinical trial data, their format and the R code used to compute various estimates are in the repository "https://github.com/KimaniPK/Treatment-Selection-with-TTE-data". The unadjusted quantities from observed data that are needed to compute various bias adjusted estimates are log HR estimates and Fisher's information obtained from interim (stage 1) analysis and naive analysis. These are given in Table S9. From stage 1 log HRs and Fisher's information, stage 1 p-values can be computed and both are less than 0.2 so that the trial continues to stage 2 with treatments 1 and 2 and with $a = 0.2$, $W_1 = -0.2948$ and $W_2 = -0.2888$.

Bias adjusted estimates are given in Table S9. From expressions for variances for estimated log HRs in Section 2.1 in the main paper, and naive estimator expression (2) (in the main paper) and UMVCUE expression (12) (in the main paper) that corresponds to the selection rule used in this example, the unbiased estimates (UMVCUEs) can be computed. This gives the unbiased log HR estimates for effects of treatments 1 and 2 as $\hat{\theta}_{1,UMV} = -0.6358$ and $\hat{\theta}_{2,UMV} = -0.5227$, respectively.

Covariance of stage 1 log hazard ratios estimates is 0.0939 (correlation is 0.7811). The joint density of stage 1 treatments 1 and 2 log HR estimates, the expression for probability of continuing to stage 2 with both treatments and expressions for the expected values for the two log HRs are given in Section 7.2 in this supplementary material. Using those expressions and from the main paper, expressions (2), (3) and (8) gives single iteration bias subtracted estimates for effects of treatments 1 and 2 as $\hat{\theta}_{1,SI} = -0.6060$ and $\hat{\theta}_{2,SI} = -0.5090$, respectively. Computation of multiple iterations bias subtracted estimates is similar, with the only difference being the inclusion of an iteration step because of using expression (9) in the main paper rather than expression (8) in the main paper. The multiple iterations bias subtracted estimates for effects of treatments 1 and 2 are $\hat{\theta}_{1,MI} = -0.5864$ and $\hat{\theta}_{2,MI} = -0.4875$, respectively.

For the shrinkage estimator, we take $\omega = 0.5$. This is informed by uniform recruitment and stage 1 patients being those recruited until the 50% point in the trial. The function to estimate ν^2 and stage 1 treatments 1 and 2 shrinkage estimates is in the supplementary. We obtain $\nu^2 = 0$ and $\hat{\theta}^{(2)}_{1,SH} = \hat{\theta}^{(2)}_{2,SH} = -0.5809$ and consequently, treatments 1 and 2 two-stage shrinkage estimates are $\hat{\theta}_{1,SH} = -0.7016$ and $\hat{\theta}_{2,SH} = -0.5889$, respectively.

The shrinkage estimates show slightly bigger treatment effects than the naive estimates (in Table S9, the naive estimates are bolded). The other bias adjusted estimates exhibit smaller effects than the naive estimates. As it would be expected in most datasets, multiple iterations bias subtracted estimates correct for biases more than the single iteration bias subtracted estimates.

Comparing Table S9 estimates to Table 3 estimates in the main paper, treatments' effects estimates are similar even though the covariances were noticeably different (0.0939 based on logrank score statistics versus 0.0522 for Cox's model).

Table S9: Worked example estimates based on logrank score statistic (**Compare with Table 3 in the main paper**)

	Cox's HR† estimates		Reconstructed data‡							
	Original	Reconstructed	Unadjusted quantities				Bias adjusted estimates			
	data	data	$\hat{\theta}_{1,j}$	$V_{1,j}$	$\hat{\theta}_j$	V_j	$\hat{\theta}_{j,UMV}$	$\hat{\theta}_{j,SI}$	$\hat{\theta}_{j,MI}$	$\hat{\theta}_{j,SH}$
Treatment 1	0.53	0.51	-0.5140	8.1488	**-0.6695**	16.4320	-0.6358	-0.6060	-0.5864	-0.7016
Treatment 2	0.63	0.56	-0.5580	8.4916	**-0.5772**	16.7772	-0.5227	-0.5090	-0.4875	-0.5889

†HR = Hazard ratio; ‡ $j = 1$ and $j = 2$ for treatments 1 and 2, respectively

.

SIMULATIONS WITH FOUR TREATMENTS IN STAGE 1
AND
ESTIMATION BASED ON LOGRANK SCORE STATISTIC

11.2 Simulation results when there are four treatments in stage 1

In this subsection, we present simulation results when there are four experimental treatments in stage 1. The results are summarised in Tables S10 and S11 and Figures S6 and S7. Comparing with previous results, except for scenario 4, the biases for the naive estimator seem to have bigger biases for Cox's model than logrank score statistics model when treatment effects in a scenario increases. This can be attributed to score statistic underestimating treatment effects when log hazard ratios are smaller than, and far from, zero (e.g. as was observed in Kimani *et al.*[8]). However, biases for most estimators that adjust for treatment selection have smaller biases with Cox's models than with logrank score statistics models (e.g. Table 4 in the main paper and Table S10 in this document).

We observed that the asymptotic distribution based on the logrank score statistic seemed to perform poorly for several simulated trials for scenarios with bigger treatments' effects, especially when selected treatment is not the most effective. This is one of the reasons that there were many simulatied trials in which there was no convergence whilst computing multiple iterations bias subtracted estimates (see Table S11). For some of these simulated trials, we could also not compute single iteration bias subtracted estimates. Consequently, the adjusted estimators did not have good properties and this is particularly obvious for scenarios with hazard ratio ≤ 0.4. For example, compare Figure S1 and Figure S7. Therefore, estimation based on the logrank score statistic is not recommended if is thought that treatments' effects are big.

Table S10: Simulated biases and root mean squared errors for the various estimators for the log hazard ratios (**Compare results with Table 4 in the main paper**)

	Selection	Simulated bias†					Root mean squared error‡				
Treatment	probability	$\hat{\theta}_j$	$\hat{\theta}_{j,UMV}$	$\hat{\theta}_{j,MI}$	$\hat{\theta}_{j,SI}$	$\hat{\theta}_{j,SH}$	$\hat{\theta}_j$	$\hat{\theta}_{j,UMV}$	$\hat{\theta}_{j,MI}$	$\hat{\theta}_{j,SI}$	$\hat{\theta}_{j,SH}$
Scenario 1: True log hazard ratios are $\theta_1 = \theta_2 = \theta_3 = \theta_4 = 0$											
1	0.2000	-0.0523	**-0.0059**	0.0148	-0.0094	-0.0342	0.0862	0.0943	0.0939	0.0834	**0.0747**
2	0.2005	-0.0527	**-0.0061**	0.0145	-0.0098	-0.0352	0.0861	0.0936	0.0935	0.0830	**0.0748**
3	0.1992	-0.0520	**-0.0051**	0.0153	-0.0089	-0.0348	0.0858	0.0937	0.0933	0.0829	**0.0748**
4	0.1995	-0.0527	**-0.0061**	0.0145	-0.0098	-0.0354	0.0860	0.0938	0.0933	0.0828	**0.0749**
Scenario 2: True log hazard ratios are $\theta_1 = \theta_2 = \theta_3 = \theta_4 = -0.2231$											
1	0.2511	-0.0386	**-0.0089**	0.0093	-0.0123	-0.0284	0.0824	0.0900	0.0903	0.0810	**0.0807**
2	0.2493	-0.0393	-0.0103	**0.0081**	-0.0132	-0.0289	0.0826	0.0891	0.0894	0.0807	**0.0806**
3	0.2517	-0.0389	-0.0090	**0.0089**	-0.0126	-0.0288	0.0826	0.0897	0.0899	0.0809	**0.0808**
4	0.2469	-0.0388	**-0.0090**	**0.0090**	-0.0124	-0.0288	0.0829	0.0902	0.0904	0.0814	**0.0812**
Scenario 3: True log hazard ratios are $\theta_1 = 0$, $\theta_2 = -0.1393$, $\theta_3 = -0.3011$ and $\theta_4 = -0.5108$											
2	0.0008	-0.0901	-0.0048	**-0.0032**	-0.0470	-0.0915	0.1095	0.0892	**0.0826**	0.0847	0.1106
3	0.0483	-0.0575	-0.0168	**-0.0049**	-0.0345	-0.0533	0.0939	0.0944	0.0900	**0.0859**	0.0932
4	0.9509	-0.0024	-0.0012	0.0025	**0.0007**	0.0087	**0.0785**	0.0798	0.0821	0.0803	0.0820
Scenario 4: True log hazard ratios are $\theta_1 = \theta_2 = \theta_3 = -0.9163$ and $\theta_4 = -1.0498$											
1	0.1035	-0.0486	-0.0390	**-0.0188**	-0.0294	-0.0915	0.0850	0.0894	0.0854	**0.0804**	0.1143
2	0.1069	-0.0493	-0.0397	**-0.0183**	-0.0299	-0.0907	0.0848	0.0884	0.0851	**0.0800**	0.1131
3	0.1046	-0.0485	-0.0389	**-0.0172**	-0.0289	-0.0900	0.0849	0.0889	0.0853	**0.0800**	0.1130
4	0.6850	**0.0004**	0.0019	0.0134	0.0109	-0.0403	**0.0722**	0.0742	0.0771	0.0733	0.0816

† Smallest bias in bold, ‡ Smallest Root mean squared error in bold

Table S11: Characteristics of simulated trials when an experimental treatment is selected. (**Compare results with Table S4 in this document.**

Treatment	Mean hazard ratios					Stage 1 deaths		Additional deaths		Non-convergent
	$\hat{\theta}_j$	$\hat{\theta}_{j,UMV}$	$\hat{\theta}_{j,MI}$	$\hat{\theta}_{j,SI}$	$\hat{\theta}_{j,SH}$	Control	Treatment j	Control	Treatment j	cases (%) for $\hat{\theta}_{j,MI}$
Scenario 1: True hazard ratios are $\exp(\theta_1) = \exp(\theta_2) = \exp(\theta_3) = \exp(\theta_4) = 1$										
1	0.95	1.00	1.02	0.99	0.97	145	135	28	30	55 (0.275)
2	0.95	1.00	1.02	0.99	0.97	145	135	28	30	65 (0.324)
3	0.95	1.00	1.02	0.99	0.97	145	135	28	30	77 (0.387)
4	0.95	1.00	1.02	0.99	0.97	145	135	28	30	68 (0.341)
Scenario 2: True hazard ratios are $\exp(\theta_1) = \exp(\theta_2) = \exp(\theta_3) = \exp(\theta_4) = 0.8$										
1	0.77	0.80	0.81	0.79	0.78	160	132	29	29	101 (0.402)
2	0.77	0.79	0.81	0.79	0.78	160	132	29	29	86 (0.345)
3	0.77	0.80	0.81	0.79	0.78	160	132	29	29	116 (0.461)
4	0.77	0.80	0.81	0.79	0.78	160	132	29	29	111 (0.450)
Scenario 3: True hazard ratios are $\exp(\theta_1) = 1$, $\exp(\theta_2) = 0.87$, $\exp(\theta_3) = 0.74$ and $\exp(\theta_4) = 0.60$										
2	0.80	0.87	0.87	0.83	0.80	160	129	29	32	7 (8.750)
3	0.70	0.73	0.74	0.72	0.70	161	122	29	29	109 (2.257)
4	0.60	0.60	0.60	0.60	0.61	161	114	29	25	8 (0.008)
Scenario 4: True hazard ratios are $\exp(\theta_1) = \exp(\theta_2) = \exp(\theta_3) = 0.40$ and $\exp(\theta_4) = 0.35$										
1	0.38	0.39	0.39	0.39	0.37	232	113	32	24	1799 (17.382)
2	0.38	0.39	0.39	0.39	0.37	232	113	32	24	1850 (17.306)
3	0.38	0.39	0.39	0.39	0.37	232	113	32	24	1779 (17.008)
4	0.35	0.35	0.36	0.35	0.34	232	109	32	21	5699 (8.320)

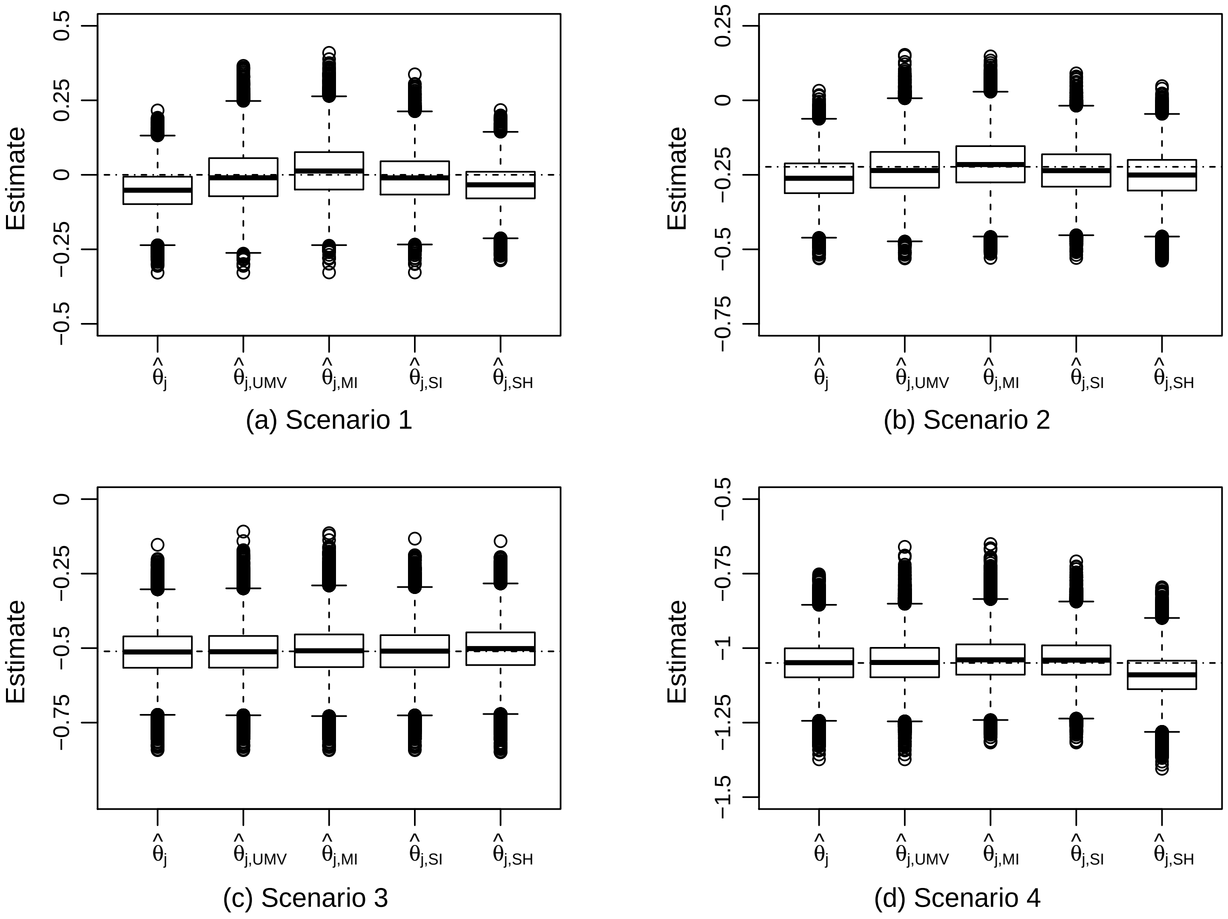

Figure S6: Boxplots for treatment four log hazard ratio estimates for scenarios 1 to 4. The true log hazard ratios are represented by the dashed and dotted lines. (**Compare with Figure 2 in the main paper**)

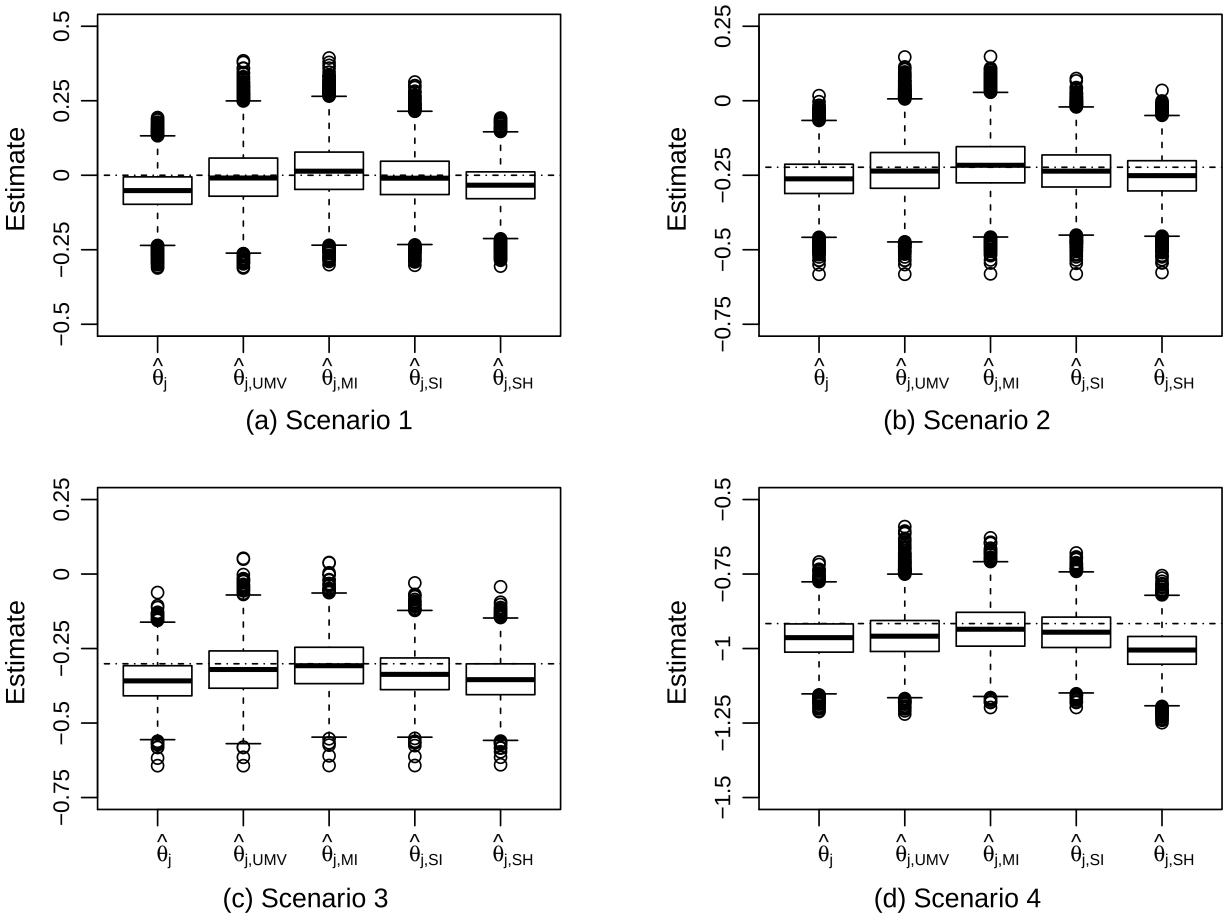

Figure S7: Boxplots for treatment three log hazard ratio estimates for scenarios 1 to 4. The true log hazard ratios are represented by the dashed and dotted lines. (**Compare with Figure S1 in this document.**)

TWO TREATMENTS IN STAGE 1
AND
ESTIMATION BASED ON LOGRANK SCORE STATISTIC

11.3 Simulation results when there are two treatments in stage 1

In this subsection, we present simulation results when there are two experimental treatments in stage 1 and estimation is based on the logrank score statistic. The results are summarised in Tables S12 and S13 and Figures S8 and S9. As is the case of comparing Cox's and logrank score estimates when there are four treatments in stage 1, comparing Cox's models results and logrank score statistics models when there are two treatments in stage 2 lead to the conclusion that in general estimation based on Cox's model is better. With the logrank score statistic, even when there is little selection bias, there is underestimation when the treatment effect is big (e.g. Figure S8(d)). Also, estimates based on logrank score statistics can perform poorly for scenarios with large treatments' effects (e.g. see Figure S9(d)).

Table S12: Simulated biases and root mean squared errors for the various estimators for the log hazard ratios for the case of two treatments in stage 1 (**Compare results with Table S5 in this document**)

Treatment	Selection probability	Simulated bias					Root mean squared error				
		$\hat{\theta}_j$	$\hat{\theta}_{j,UMV}$	$\hat{\theta}_{j,MI}$	$\hat{\theta}_{j,SI}$	$\hat{\theta}_{j,SH}$	$\hat{\theta}_j$	$\hat{\theta}_{j,UMV}$	$\hat{\theta}_{j,MI}$	$\hat{\theta}_{j,SI}$	$\hat{\theta}_{j,SH}$
Scenario 1: True log hazard ratios are $\theta_1 = \theta_2 = 0$											
1	0.3353	-0.0443	**-0.0047**	0.0096	-0.0084	-0.0363	0.0796	0.0905	0.0901	0.0809	**0.0731**
2	0.3339	-0.0444	**-0.0048**	0.0096	-0.0085	-0.0368	0.0793	0.0899	0.0895	0.0805	**0.0731**
Scenario 2: True log hazard ratios are $\theta_1 = \theta_2 = -0.2231$											
1	0.4986	-0.0216	**-0.0057**	0.0058	-0.0073	-0.0155	0.0758	0.0828	0.0848	0.0781	**0.0755**
2	0.4965	-0.0216	-0.0059	**0.0058**	-0.0072	-0.0156	0.0762	0.0832	0.0851	0.0785	**0.0758**
Scenario 3: True log hazard ratios are $\theta_1 = -0.3011$ and $\theta_2 = -0.5108$											
1	0.0447	-0.0564	-0.0161	**-0.0024**	-0.0347	-0.0678	0.0911	0.0905	0.0882	**0.0828**	0.1010
2	0.9553	-0.0014	**-0.0003**	0.0029	0.0013	0.0023	**0.0756**	0.0767	0.0787	0.0771	0.0788
Scenario 4: True log hazard ratios are $\theta_1 - 0.9163$ and $\theta_2 = -1.0498$											
1	0.1636	-0.0331	-0.0234	**-0.0022**	-0.0204	-0.0682	0.0797	0.0849	0.0882	**0.0787**	0.0993
2	0.8364	**0.0146**	0.0155	0.0197	0.0175	-0.0155	0.0761	0.0774	0.0809	0.0779	**0.0757**

Table S13: Additional information of the simulation study for the case of two treatments in stage 1 (**Compare results with Table S6 in this document**)

Treatment	Mean hazard ratios					Stage 1 deaths		Additional deaths		Non-convergent
	$\hat{\theta}_j$	$\hat{\theta}_{j,UMV}$	$\hat{\theta}_{j,MI}$	$\hat{\theta}_{j,SI}$	$\hat{\theta}_{j,SH}$	Control	Treatment j	Control	Treatment j	cases for $\hat{\theta}_{j,MI}$
Scenario 1: True hazard ratios are $\exp(\theta_1) = \exp(\theta_2) = 1$										
1	0.96	1.00	1.01	0.99	0.97	149	139	39	42	2 (0.006)
2	0.96	1.00	1.01	0.99	0.97	149	139	39	42	1 (0.003)
Scenario 2: True hazard ratios are $\exp(\theta_1) = \exp(\theta_2) = 0.8$										
1	0.78	0.80	0.81	0.80	0.79	161	134	41	39	0 (0)
2	0.78	0.80	0.81	0.80	0.79	161	133	41	39	0 (0)
Scenario 3: True hazard ratios are $\exp(\theta_1) = 0.74$ and $\exp(\theta_2) = 0.60$										
1	0.70	0.73	0.74	0.72	0.69	174	129	42	40	0 (0)
2	0.60	0.60	0.60	0.60	0.60	174	121	43	35	0 (0)
Scenario 4: True hazard ratios are $\exp(\theta_1) = 0.40$ and $\exp(\theta_2) = 0.35$										
1	0.39	0.39	0.40	0.39	0.37	221	105	29	22	417 (2.549)
2	0.36	0.36	0.36	0.36	0.35	220	101	29	19	533 (0.637)

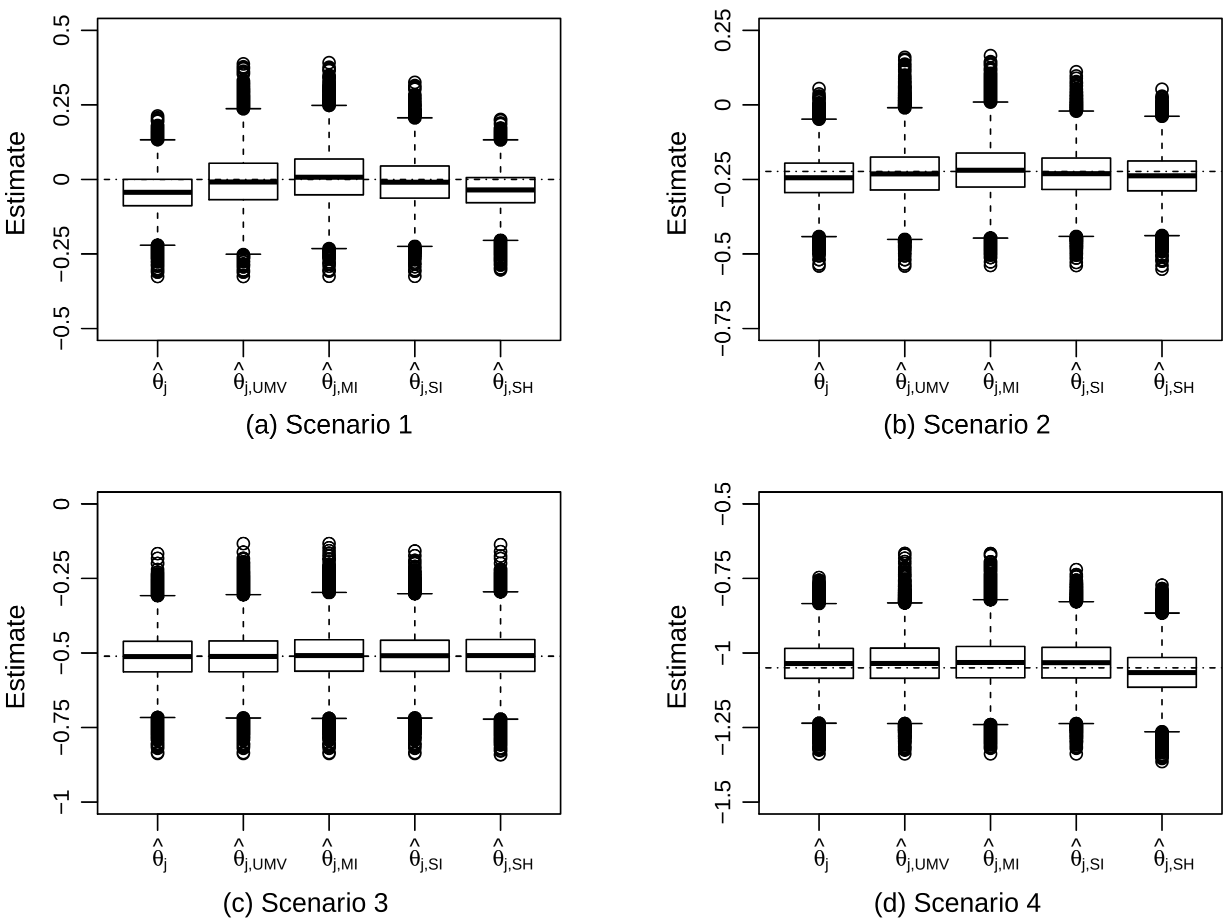

Figure S8: Boxplots for treatment two log hazard ratio estimates for scenarios 1 to 4 for the case of two experimental treatments in stage 1. The true log hazard ratios are represented by the dashed and dotted lines. (**Compare results with Figure S2 in this document**)

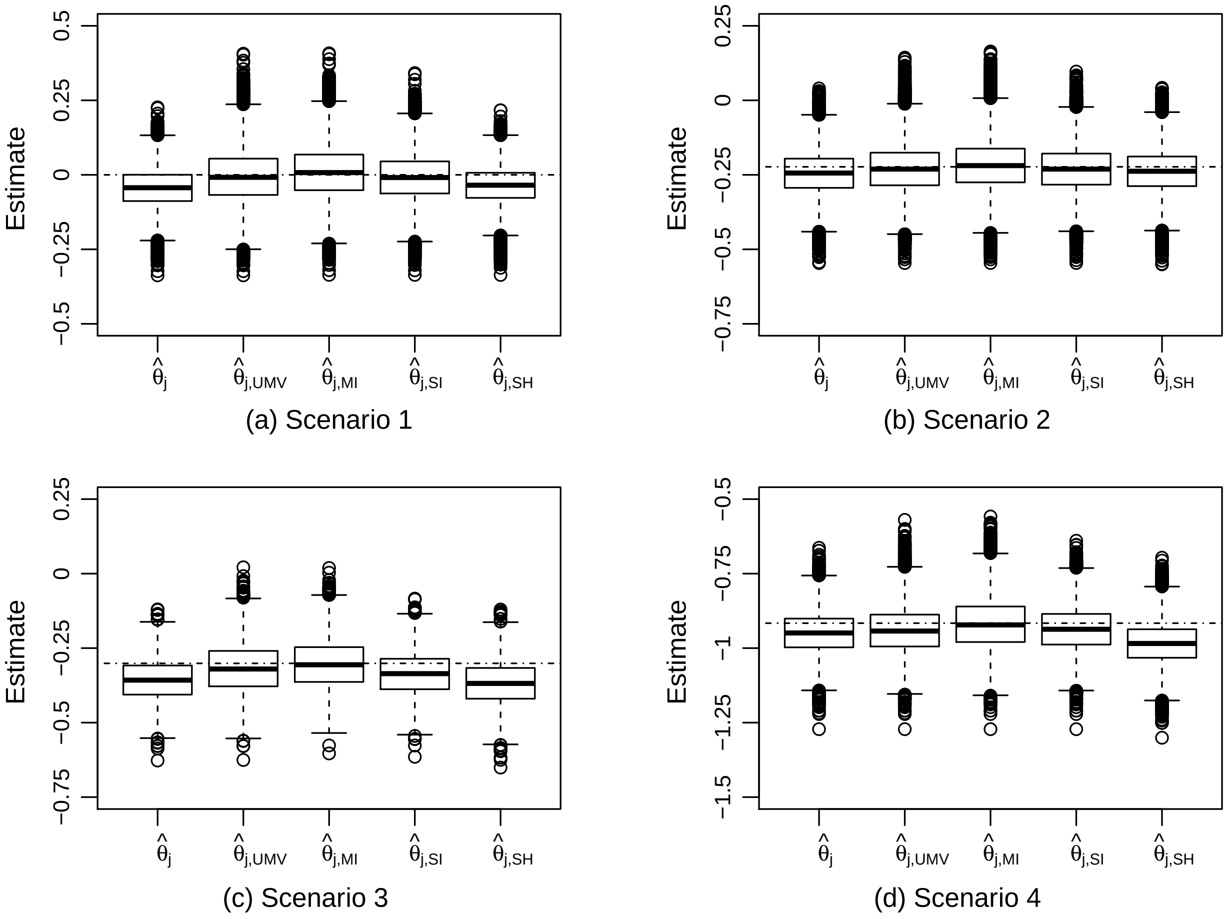

Figure S9: Boxplots for treatment one log hazard ratio estimates for scenarios 1 to 4 for the case of two experimental treatments in stage 1. The true log hazard ratios are represented by the dashed and dotted lines. (**Compare results with Figure S3 in this document**)

SIMULATIONS WITH A SMALLER SAMPLE SIZE
AND
FOUR TREATMENTS IN STAGE ONE

11.4 Simulation results for the case of smaller sample size and four treatments in stage 1

In this subsection we present simulation results when there are four experimental treatments in stage 1. Comparing with previous results, except for scenario 4, the biases for the naive estimator seem to have bigger biases for Cox's model than logrank score statistics model when treatment effects in a scenario increases. This can be attributed to score statistic underestimating treatment effects when log hazard ratios are smaller than and far from zero (e.g. as was observed in Kimani *et al.*[8]). However, biases for the estimators that adjust for treatment effects have smaller biases with Cox's models than with logrank score statistics models. This difference starts to get obvious and of practible use when hazard ratio is 0.4.

Table S14: Simulated biases and root mean squared errors for the various estimators for the log hazard ratios (**Compare results with Table 4 in the main paper**)

Treatment	Selection probability	Simulated bias† $\hat{\theta}_j$	$\hat{\theta}_{j,UMV}$	$\hat{\theta}_{j,MI}$	$\hat{\theta}_{j,SI}$	$\hat{\theta}_{j,SH}$	Root mean squared error‡ $\hat{\theta}_j$	$\hat{\theta}_{j,UMV}$	$\hat{\theta}_{j,MI}$	$\hat{\theta}_{j,SI}$	$\hat{\theta}_{j,SH}$
Scenario 1: True log hazard ratios are $\theta_1 = \theta_2 = \theta_3 = \theta_4 = 0$											
1	0.1994	-0.1051	-0.0218	0.0193	**-0.0191**	-0.0773	0.1808	0.1934	0.1909	0.1760	**0.1611**
2	0.2022	-0.1036	-0.0204	0.0209	**-0.0174**	-0.0815	0.1791	0.1917	0.1893	0.1744	**0.1614**
3	0.1990	-0.1041	-0.0205	0.0209	**-0.0177**	-0.0828	0.1808	0.1939	0.1920	0.1766	**0.1637**
4	0.2011	-0.1035	-0.0197	0.0216	**-0.0171**	-0.0832	0.1796	0.1933	0.1909	0.1753	**0.1634**
Scenario 2: True log hazard ratios are $\theta_1 = \theta_2 = \theta_3 = \theta_4 = -0.2231$											
1	0.2417	-0.0830	-0.0263	**0.0145**	-0.0203	-0.0617	0.1723	0.1865	0.1865	0.1727	**0.1695**
2	0.2410	-0.0828	-0.0260	**0.0149**	-0.0200	-0.0643	0.1724	0.1865	0.1868	0.1729	**0.1687**
3	0.2422	-0.0841	-0.0268	**0.0133**	-0.0213	-0.0660	0.1733	0.1884	0.1877	0.1738	**0.1696**
4	0.2445	-0.0842	-0.0277	**0.0129**	-0.0216	-0.0666	0.1734	0.1879	0.1878	0.1740	**0.1697**
Scenario 3: True log hazard ratios are $\theta_1 = 0$, $\theta_2 = -0.1393$, $\theta_3 = -0.3011$ and $\theta_4 = -0.5108$											
1	0.0119	-0.1546	-0.0314	**0.0040**	-0.0576	-0.1641	0.2119	0.1926	0.1830	**0.1774**	0.2218
2	0.0551	-0.1216	-0.0338	**0.0037**	-0.0459	-0.1160	0.1943	0.1966	0.1881	**0.1785**	0.1940
3	0.2066	-0.0814	-0.0299	**0.0059**	-0.0285	-0.0629	0.1747	0.1882	0.1872	0.1750	**0.1736**
4	0.7148	-0.0286	-0.0142	0.0103	**-0.0019**	0.0088	**0.1615**	0.1724	0.1814	0.1727	0.1710
Scenario 4: True log hazard ratios are $\theta_1 = \theta_2 = \theta_3 = -0.9163$ and $\theta_4 = -1.0498$											
1	0.1863	-0.0776	-0.0583	**-0.0145**	-0.0339	-0.1713	0.1614	0.1715	0.1694	**0.1569**	0.2278
2	0.1897	-0.0763	-0.0571	**-0.0130**	-0.0321	-0.1658	0.1617	0.1721	0.1708	**0.1583**	0.2237
3	0.1886	-0.0767	-0.0577	**-0.0132**	-0.0330	-0.1668	0.1623	0.1723	0.1718	**0.1587**	0.2248
4	0.4355	-0.0286	-0.0195	0.0148	**0.0044**	-0.1114	**0.1465**	0.1556	0.1629	0.1513	0.1888

† Smallest bias in bold, ‡ Smallest Root mean squared error in bold

Table S15: Characteristics of simulated trials when an experimental treatment is selected. (**Compare results with Table S4 in this document.**

Treatment	Mean hazard ratios					Stage 1 deaths		Additional deaths		Non-convergent
	$\hat{\theta}_j$	$\hat{\theta}_{j,UMV}$	$\hat{\theta}_{j,MI}$	$\hat{\theta}_{j,SI}$	$\hat{\theta}_{j,SH}$	Control	Treatment j	Control	Treatment j	cases (%) for $\hat{\theta}_{j,MI}$
Scenario 1: True hazard ratios are $\exp(\theta_1) = \exp(\theta_2) = \exp(\theta_3) = \exp(\theta_4) = 1$										
1	0.91	1.00	1.04	1.00	0.93	30	25	15	17	63 (0.316)
2	0.91	1.00	1.04	1.00	0.93	30	25	15	17	59 (0.292)
3	0.91	1.00	1.04	1.00	0.93	30	25	15	17	76 (0.382)
4	0.91	1.00	1.04	1.00	0.93	30	25	15	17	76 (0.378)
Scenario 2: True hazard ratios are $\exp(\theta_1) = \exp(\theta_2) = \exp(\theta_3) = \exp(\theta_4) = 0.8$										
1	0.74	0.79	0.83	0.80	0.76	34	24	16	16	120 (0.496)
2	0.74	0.79	0.83	0.80	0.76	34	24	16	16	133 (0.552)
3	0.74	0.79	0.83	0.79	0.76	34	24	16	16	149 (0.615)
4	0.74	0.79	0.82	0.79	0.76	34	24	16	16	133 (0.544)
Scenario 3: True hazard ratios are $\exp(\theta_1) = 1$, $\exp(\theta_2) = 0.87$, $\exp(\theta_3) = 0.74$ and $\exp(\theta_4) = 0.60$										
1	0.87	0.99	1.02	0.96	0.86	34	25	16	19	30 (2.521)
2	0.78	0.86	0.89	0.84	0.78	34	24	16	17	94 (1.706)
3	0.69	0.73	0.76	0.73	0.70	34	22	16	15	204 (0.987)
4	0.59	0.60	0.62	0.61	0.61	34	21	17	13	111 (0.155)
Scenario 4: True hazard ratios are $\exp(\theta_1) = \exp(\theta_2) = \exp(\theta_3) = 0.40$ and $\exp(\theta_4) = 0.35$										
1	0.37	0.38	0.40	0.39	0.34	51	20	20	12	2217 (11.900)
2	0.37	0.38	0.40	0.39	0.34	51	20	20	12	2252 (11.871)
3	0.37	0.38	0.40	0.39	0.34	51	20	20	12	2195 (11.638)
4	0.34	0.35	0.36	0.36	0.32	51	19	20	11	4406 (10.117)

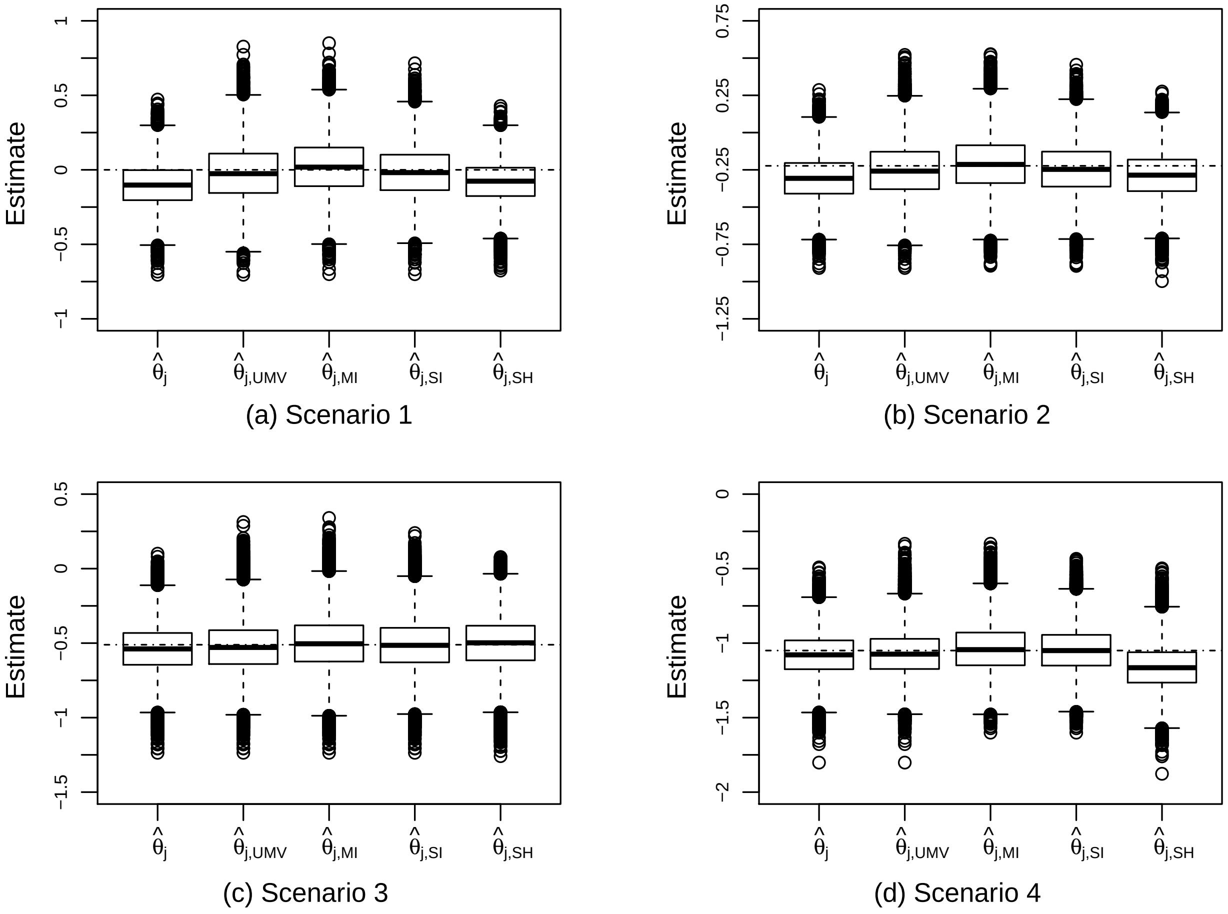

Figure S10: Boxplots for treatment four log hazard ratio estimates for scenarios 1 to 4. The true log hazard ratios are represented by the dashed and dotted lines. (**Compare with Figure 2 in the main paper**)

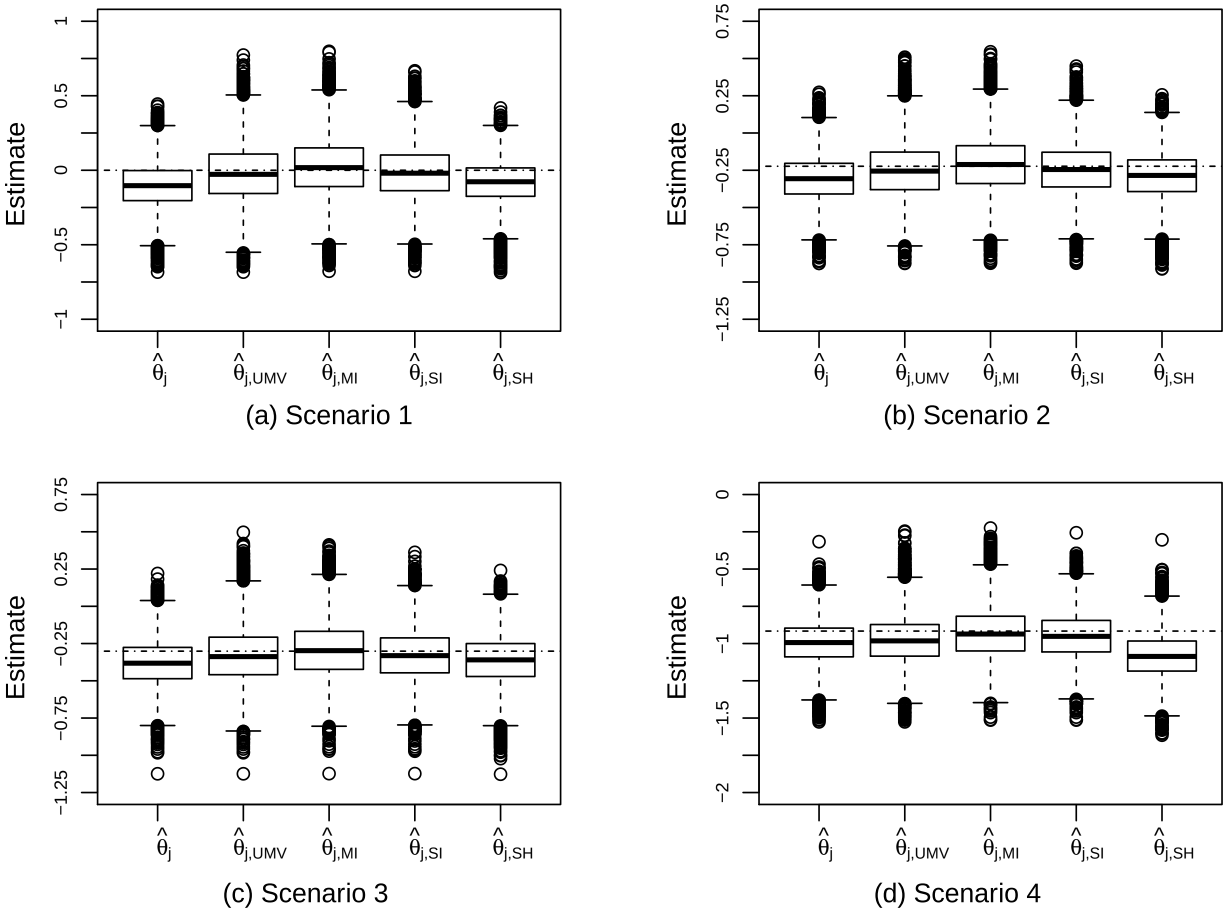

Figure S11: Boxplots for treatment three log hazard ratio estimates for scenarios 1 to 4. The true log hazard ratios are represented by the dashed and dotted lines. (**Compare with Figure S1 in this document.**)

References

[1] Jack Bowden and Ekkehard Glimm. Unbiased estimation of selected treatment means in two-stage trials. *Biometrical Journal*, **50**(4):515 – 527, 2008.

[2] Peter K Kimani, Susan Todd, Lindsay A Renfro, and Nigel Stallard. Point estimation following two-stage adaptive threshold enrichment clinical trials. *Statistics in Medicine*, **37**:3179–3196, 2018.

[3] Raymond Kan and Cesare Robotti. On moments of folded and truncated multivariate normal distributions. *Journal of Computational and Graphical Statistics*, **26**(4):930–934, 2017.

[4] Matthias Brückner, Andrew Titman, and Thomas Jaki. Estimation in multi-arm two-stage trials with treatment selection and time-to-event endpoint. *Statistics in Medicine*, **36**:3137–3153, 2017.

[5] Peter Bauer, Franz Koenig, Werner Brannath, and Martin Posch. Selection and bias - two hostile brothers. *Statistics in Medicine*, **29**:1 – 13, 2010.

[6] Máximo Carreras and Werner Brannath. Shrinkage estimation in two-stage adaptive designs with mid-trial treatment selection. *Statistics in Medicine*, **32**(10):1677 – 1690, May 2013.

[7] Peter K Kimani, Susan Todd, and Nigel Stallard. Conditionally unbiased estimation in phase II/III clinical trials with early stopping for futility. *Statistics in Medicine*, **32**(17):2893–2910, July 2013.

[8] Peter K Kimani, Susan Todd, Lindsay A Renfro, Ekkehard Glimm, Josephine N Khan, John A Kairalla, and Nigel Stallard. Point and interval estimation in two-stage adaptive designs with time to event data and biomarker-driven subpopulation selection. *Statistics in Medicine*, **39**:2568–2586, 2020.
